# Pyonephrosis—An Updated Morphological Glimpse into the Contemporary Management of an Immemorial Pathology: A Narrative Review

**DOI:** 10.3390/diagnostics16203334

**Published:** 2026-10-14

**Authors:** Silviu Latcu, Dorin Novacescu, Adelina Baloi, Flavia Zara, Cristina-Stefania Dumitru, Radu Caprariu, Talida Georgiana Cut, Adelina Marinescu, Voichita Elena Lazureanu, Felix Bratosin, Flaviu Lucian Petrica, Alin Cumpanas

**Affiliations:** 1 Bastion Policlinic, Mircea cel Batran Street, No. 122C, 300600 Timisoara, Romania; silviu.latcu@umft.ro (S.L.); medical@policlinicabastion.ro (F.L.P.); 2 Doctoral School, Victor Babes University of Medicine and Pharmacy Timisoara, E. Murgu Square, No. 2, 300041 Timisoara, Romania; 3 Department XV, Discipline of Urology, Victor Babes University of Medicine and Pharmacy Timisoara, E. Murgu Square, No. 2, 300041 Timisoara, Romania; cumpanas.alin@umft.ro; 4 Department II of Microscopic Morphology, Victor Babes University of Medicine and Pharmacy Timisoara, E. Murgu Square, No. 2, 300041 Timisoara, Romaniacristina-stefania.dumitru@umft.ro (C.-S.D.); 5 Department X—Surgery II, Discipline Anesthesia and Intensive Care, Victor Babes University of Medicine and Pharmacy Timisoara, E. Murgu Square, Nr. 2, 300041 Timisoara, Romania; adelina.baloi@umft.ro; 6 Department XV, Discipline of Radiology and Medical Imaging, Victor Babes University of Medicine and Pharmacy Timisoara, E. Murgu Square, Nr. 2, 300041 Timisoara, Romania; 7 Department XIII, Discipline of Infectious Diseases, Victor Babes University of Medicine and Pharmacy Timisoara, E. Murgu Square, No. 2, 300041 Timisoara, Romania; talida.cut@umft.ro (T.G.C.); adelina.marinescu@umft.ro (A.M.); lazureanu.voichita@umft.ro (V.E.L.); 8 Division of Infectious Diseases, Department of Internal Medicine, McGovern Medical School, UTHealth Houston, Houston, TX 77030, USA; felix.bratosin@uth.tmc.edu

**Keywords:** pyonephrosis, acute kidney injury, chronic kidney disease, infected hydronephrosis, urosepsis, percutaneous nephrostomy, Hounsfield unit, diagnostic imaging

## Abstract

Pyonephrosis—pus within an obstructed, infected renal collecting system, with suppurative parenchymal destruction—is a urological emergency and a potentially reversible cause of acute kidney injury (AKI); a contribution to chronic kidney disease (CKD) is biologically plausible but has not been demonstrated in pyonephrosis-specific longitudinal studies. This narrative review, reported with reference to the SANRA criteria and based on structured searches of PubMed/MEDLINE, Scopus, and Web of Science, synthesizes the epidemiology, pathophysiology, microbiology, morphological diagnosis, and contemporary management of pyonephrosis, distinguishing evidence generated in confirmed pyonephrosis from that in infected hydronephrosis, obstructive pyelonephritis, and stone-associated sepsis. Sonographic, computed tomographic, and magnetic resonance features are illustrated with original schematics and a case of perforated calculous pyonephrosis; reported Hounsfield-unit thresholds and apparent diffusion coefficients derive from small, single-center cohorts and are protocol- and population-dependent rather than universal cut-offs. Urgent decompression is strongly recommended by guidelines, but the supporting data are observational, so associations between drainage, its timing, and mortality remain vulnerable to confounding. Randomized comparisons of percutaneous nephrostomy and retrograde stenting are few, small, and inconsistent, and establish neither superiority nor equivalence in infected systems. We rate the certainty of each principal conclusion and identify research priorities.

## 1. Introduction

The kidneys’ dual role in fluid homeostasis and solute clearance means that any process that obstructs and infects the collecting system threatens the affected organ acutely and may plausibly also contribute to chronic kidney disease (CKD), which now affects an estimated 14.2% of the world’s adult population (aged 20 years and older) [1]. Few conditions illustrate an acute threat as directly, or as urgently, as pyonephrosis. Early twentieth-century histological examination of rehydrated tissue from Egyptian mummies of the Eighteenth to Twentieth Dynasties described renal abscesses containing stainable bacteria [2,3,4] (albeit a morphological observation that cannot be equated with a microbiological diagnosis; see Section 3), and the Ebers and related papyri already contained remedies for urinary retention, hematuria, and the “sending forth of heat from the bladder” more than three millennia ago [2,5]—a testament to how long suppurative kidney disease has troubled human populations, and the reason we describe pyonephrosis in this review’s title as an immemorial pathology. And yet, despite this ancient pedigree, pyonephrosis is also a thoroughly modern problem: it is a condition whose diagnosis now rests on quantitative cross-sectional imaging and whose management has been reshaped, within the last seventy years, from an era of high-mortality open surgery to one of same-day, image-guided percutaneous drainage.

Pyonephrosis is defined as infected hydronephrosis with suppurative destruction of the renal parenchyma and consequent loss of renal function—colloquially, “pus under pressure” within an obstructed collecting system [6,7]. It is a true urological emergency and a classical, potentially reversible cause of obstructive acute kidney injury (AKI): unrelieved, it can progress within hours to urosepsis and septic shock, and, in administrative cohorts of stone-associated infection and sepsis, absent or delayed decompression has been associated with higher in-hospital mortality [8,9]. Equally relevant for the kidney disease community more broadly, the parenchymal destruction that defines pyonephrosis—if the diagnosis is delayed, if drainage is incomplete, or if episodes recur—is not necessarily fully reversible. A link between pyonephrosis and subsequent CKD is therefore biologically plausible; it has not, however, been demonstrated with pyonephrosis-specific longitudinal data and is treated throughout this review as a hypothesis rather than an established outcome (see Section 13). Because obstruction and infection first accumulate silently within the collecting system before overt sepsis develops, and because bladder or midstream urine culture often fails to reflect infection above the obstruction [10]—it missed the upper-tract pathogen in roughly one-third to two-thirds of patients in small series of obstructed, infected kidneys [11,12,13]—the diagnosis of pyonephrosis is fundamentally a morphological one: it is imaging—ultrasonography, computed tomography (CT), and increasingly magnetic resonance imaging (MRI)—that most often distinguishes pyonephrosis from simple, sterile hydronephrosis and triggers the emergency drainage on which both septic and renal-functional outcomes depend.

This narrative review has two complementary aims, both signaled in its title. The first is to provide an updated morphological account of pyonephrosis: the sonographic, tomographic, and magnetic-resonance features that characterize the disease, the quantitative thresholds—attenuation values on non-contrast CT and apparent diffusion coefficients on diffusion-weighted MRI (DWI-MRI)—that have been proposed to formalize what was once a purely qualitative radiological impression, and the pitfalls, illustrated schematically and with imaging from our own clinical practice, that make this differentiation more difficult than textbook descriptions sometimes suggest. The second is to synthesize contemporary clinical management, from initial resuscitation and recognition of urosepsis, through the central and still-debated choice between percutaneous nephrostomy (PCN) and retrograde ureteral stenting for urgent decompression, to the later, deliberately delayed treatment of the underlying obstructive cause and the indications for nephrectomy in the destroyed, non-functioning kidney. Throughout, we set this contemporary evidence against its historical background, tracing how a disease once treated—when it was treated at all—by open surgery or left to its frequently fatal natural history came to be managed by needle and catheter. We close by considering recent advances, including radiomic and machine-learning prediction models, and by identifying where the evidence base, particularly regarding the optimal drainage modality, remains genuinely unsettled. To that end, we specify the population in which each piece of evidence was generated—confirmed pyonephrosis, infected hydronephrosis, obstructive pyelonephritis, or stone-associated sepsis (see Section 4)—and we rate the certainty of each principal conclusion (see Section 13).

## 2. Materials and Methods

### 2.1. Review Design and Reporting

This article is a narrative review. It is not a systematic review: no protocol was registered, no quantitative synthesis was undertaken, and the PRISMA statement is therefore not applicable. The review is reported with reference to the Scale for the Assessment of Narrative Review Articles (SANRA), whose six items—justification of the article’s importance for the readership, statement of concrete aims, description of the literature search, referencing, scientific reasoning, and appropriate presentation of data—were used as a reporting checklist [14].

### 2.2. Information Sources and Search Strategy

The literature was identified through searches of PubMed/MEDLINE, Scopus, and Web of Science, each covering the period from database inception to the date of the last search (15 August 2026). The search terms were “pyonephrosis”, “infected hydronephrosis”, “obstructive pyelonephritis”, “urosepsis”, “percutaneous nephrostomy”, “ureteral stent”, “Hounsfield unit”, and “xanthogranulomatous pyelonephritis”. They were entered as quoted phrases in the title, abstract, and keyword fields of each database (PubMed: Title/Abstract; Scopus: TITLE-ABS-KEY; Web of Science: Topic) and combined with Boolean operators into the following string, adapted to the syntax of each database: (“pyonephrosis” OR “infected hydronephrosis” OR “obstructive pyelonephritis” OR “xanthogranulomatous pyelonephritis”) OR (“urosepsis” AND (“percutaneous nephrostomy” OR “ureteral stent” OR “Hounsfield unit”)). No filters for publication date, article type, or language were applied at the search stage; the eligibility criteria in Section 2.3, including the restriction to English-language sources, were applied during screening. The reference lists of retrieved articles and of current international guidelines were hand-searched for additional and historically significant sources.

### 2.3. Eligibility Criteria

Sources were eligible if they met the following criteria. Population: adults or children with pyonephrosis or with one of the adjacent obstructed, infected upper-tract conditions defined in Section 4 (infected hydronephrosis, obstructive pyelonephritis, or stone-associated sepsis), provided that the population category could be identified. Definition of pyonephrosis: for statements on diagnostic accuracy and outcome, priority was given to studies in which pyonephrosis was confirmed by frankly purulent fluid at aspiration, drainage, or surgery, with or without a positive collecting-system culture; studies that relied on clinical or radiological definitions were included but are labeled as such. Index tests: ultrasonography, CT (including attenuation measurement), MRI (including diffusion-weighted imaging), radionuclide studies, and blood or urinary biomarkers. Interventions and comparators: PCN, retrograde ureteral stenting or catheterization, early definitive stone treatment, nephrectomy, and antimicrobial therapy, compared with one another or with delayed or no decompression. Outcomes: diagnostic accuracy against the reference standard used in each study, technical success and rescue procedures, time to clinical recovery, septic complications, mortality, renal function, and quality of life. Study types: randomized and non-randomized comparative studies, cohort, case–control, cross-sectional, and diagnostic-accuracy studies, case series, systematic reviews and meta-analyses, and current international guidelines; single case reports were used only to illustrate rare complications, and historical or paleopathological sources were used only for Section 3. Language: English, with the exception of historically significant original descriptions. Conference abstracts without a full-text publication, duplicate reports of the same cohort, and animal studies other than established experimental models of ureteral obstruction (cited for mechanistic context only) were excluded.

### 2.4. Study Selection and Data Extraction

Two authors (D.N. and R.C.) independently screened titles and abstracts and then assessed the full texts of potentially relevant records against the eligibility criteria; records retrieved from more than one database were de-duplicated with reference-management software and by manual checking before screening. For each included source, the same two authors extracted the study design, setting, sample size, population category, definition of or reference standard for pyonephrosis, index test or intervention, comparator, and outcome data—including effect estimates and 95% confidence intervals where reported—into a standardized spreadsheet. Disagreements at any stage were resolved by discussion and, where necessary, by a third, senior author (S.L.). In keeping with the narrative design, the selection process was not documented in a flow diagram, and the final selection reflects the authors’ judgment of clinical relevance within these criteria.

### 2.5. Appraisal of the Evidence and Certainty of Conclusions

No formal study-level risk-of-bias scoring was performed. Instead, the key studies underpinning each principal conclusion were appraised qualitatively with attention to the domains of the design-appropriate instruments—QUADAS-2 for diagnostic-accuracy studies (patient selection, index test, reference standard, and flow and timing) [15], RoB 2 for randomized trials [16], and ROBINS-I for non-randomized comparisons of interventions (in particular confounding, selection of participants, and classification of interventions) [17]—and the certainty of each conclusion was rated by consensus as high, moderate, low, or very low. These ratings were informed by the GRADE domains (study limitations, inconsistency, indirectness, imprecision, and publication bias) [18], with indirectness taken to include evidence generated in a population adjacent to, rather than restricted to, confirmed pyonephrosis; they are the authors’ judgments, not the product of a formal GRADE assessment, and are presented in Section 13. Effect estimates are reported with 95% confidence intervals wherever the source provides them, and their absence is stated; findings from observational studies are described as associations.

## 3. Historical Perspective: From an Immemorial Affliction to a Defined Clinical Entity

The kidney’s propensity to suppurate under obstruction long predates its clinical description. The paleopathological evidence is suggestive rather than conclusive. In 1910, Ruffer reported that renal abscesses were recognizable in organs from Egyptian mummies of the Eighteenth to Twentieth Dynasties and that, among six mummies whose kidneys he examined, those of one contained multiple abscesses with well-staining bacteria; he noted that micro-organisms in these lesions could be stained with methylene blue, fuchsin, hematoxylin, and Gram’s method [3]. A later summary described the organisms as bacilli that were most probably colon bacilli and dated the specimen to the Eighteenth Dynasty [4], whereas a widely cited nephrological review refers to pyonephrosis and multiple abscesses in both kidneys due to Gram-negative coccobacilli in mummies of the “28th dynasty” [2]; neither that dynasty, nor the diagnosis of pyonephrosis, nor a Gram-negative coccobacillary morphology appears in Ruffer’s note. Because the observation rests on a single mummy and on the morphology of organisms in desiccated, rehydrated tissue, in which post-mortem bacterial proliferation cannot be excluded, and because it has never been corroborated by molecular methods, it shows that suppurative renal disease existed in pharaonic Egypt but establishes neither the causative organism nor the presence of obstruction or of pyonephrosis in the modern sense. Urinary calculi have likewise been recovered from ancient Egyptian remains, including three described by Ruffer that reportedly contained leukocytes, epithelial cells, and a mixture of pus and uric acid [2,5]. Ancient Egyptian medical papyri already catalogued remedies for hematuria, retention, and urinary infection, and comparably early textual traditions—the Mesopotamian Asûtu texts, the Sanskrit surgical corpus of Suśruta, and the Hippocratic corpus—independently record the symptoms and, in some cases, surgical approaches to urinary stone disease, the principal cause of pyonephrosis today as in antiquity [5]. For most of this long history, however, an infected, obstructed kidney was a lethal or disfiguring condition managed, at best, by open incision and drainage or nephrectomy, and diagnosis rested entirely on palpation, percussion, and the appearance of pus at surgery.

The modern era of image-guided, minimally invasive management began, somewhat improbably, in 1865, when the British physician Thomas Hillier repeatedly punctured and aspirated a massively hydronephrotic kidney in a four-year-old boy with ureteropelvic junction obstruction, achieving temporary decompression well before the advent of radiography or antisepsis [19]. Radiographic guidance had to await the description of antegrade pyelography, after which Willard Goodwin, Casey, and Woolf formalized percutaneous trocar (needle) nephrostomy as a reproducible technique for the temporary drainage of hydronephrosis in 1955—the paper that is conventionally credited with founding modern percutaneous nephrostomy [20]. Complementary endourological milestones followed over the subsequent two decades: Rupel and Brown had already, in 1941, performed the first nephroscopy by passing a rigid cystoscope through a nephrostomy tract during open surgery for obstructive calculous anuria [21]; Fernström and Johansson described percutaneous extraction of renal calculi through a mature nephrostomy tract in 1976 [22]; and Arthur Smith introduced antegrade ureteral stent placement facilitated by an antecedent percutaneous nephrostomy in 1978, work that helped establish the discipline subsequently named “endourology” [23]. Together, these developments converted percutaneous nephrostomy from an occasional, improvised maneuver into a standardized emergency procedure, and set the technical foundation on which the randomized comparisons between percutaneous and retrograde drainage discussed later in this review would eventually be built [24,25].

The consequence of this century-long trajectory is that the outlook of pyonephrosis appears to have been transformed by technology rather than by antimicrobial therapy alone, although the supporting evidence is observational: in a national inpatient cohort of septic patients with ureteral calculi (stone-associated sepsis rather than confirmed pyonephrosis), in-hospital mortality was 8.82% among those who underwent decompression, compared with 19.2% among those who did not [8]—an association that cannot be read as the causal effect of drainage (see Section 11)—whereas the mortality of approximately 50% sometimes quoted for obstruction combined with infection [6] derives from general sepsis epidemiology rather than from pyonephrosis-specific series. The disease has not changed; the speed and precision with which it can now be recognized and decompressed—and, in all likelihood, the odds of preserving life and renal function—have.

## 4. Definition, Nomenclature, and Disease Spectrum

Pyonephrosis denotes infected hydronephrosis in which pus and inflammatory debris accumulate under pressure within an obstructed pelvicalyceal system, producing suppurative destruction of the adjacent renal parenchyma and partial or total loss of function in the affected kidney [6]. The term derives from the Greek pyon (pus) and nephros (kidney), and its use in the literature has long been complicated by inconsistent boundaries with five related entities:

- Acute pyelonephritis refers to parenchymal bacterial infection, typically ascending, in a collecting system that is not significantly obstructed and does not contain frank pus under pressure. Pyonephrosis can be conceptualized as arising along the same ascending-infection pathway as pyelonephritis, but distinguished by the coexistence of mechanical obstruction and a purulent, non-draining reservoir;
- Infected hydronephrosis (sometimes used synonymously with pyonephrosis in the literature, and sometimes as a milder point on the same continuum) denotes bacteriuria or bacterial colonization of a dilated collecting system without the gross purulence and parenchymal destruction implied by “pyonephrosis” proper; many authors reserve the latter term for the more severe, frankly suppurative end of this spectrum;
- Obstructive pyelonephritis (also termed obstructive acute pyelonephritis) is a clinical diagnosis: upper-tract obstruction, most often by a ureteral calculus, accompanied by fever, leukocytosis, or other signs of infection. The contents of the collecting system are not sampled as part of the definition, so the category includes both infected hydronephrosis and unrecognized pyonephrosis; most randomized and prospective studies of urgent drainage enrolled patients defined in this way;
- Stone-associated sepsis (obstructive urosepsis or infected urolithiasis) refers to sepsis or coded urinary infection in a patient with an obstructing stone, as identified from diagnosis codes in administrative datasets; neither the contents of the collecting system nor, as a rule, clinical severity are recorded, and the proportion of such patients with true pyonephrosis is unknown;
- Renal or perinephric abscess describes a focal, typically encapsulated suppurative collection within the parenchyma or perinephric space, which may occur with or without associated pelvicalyceal obstruction and is managed somewhat differently (targeted percutaneous or surgical drainage of the abscess cavity itself) than the diffuse, pressurized, obstructed collecting system of pyonephrosis.

Two further, closely related entities occupy the more severe or chronic end of the same disease spectrum and are discussed in detail below: xanthogranulomatous pyelonephritis (XGP), a rare, chronic, destructive granulomatous variant most often associated with obstructing staghorn calculi, and emphysematous pyelonephritis (EPN), a necrotizing, gas-forming infection that shares pyonephrosis’ obstructive-suppurative mechanism but is defined radiologically by the presence of gas within the renal parenchyma or collecting system. Both are, in effect, extreme morphological phenotypes of the same obstructive–suppurative spectrum discussed throughout this review: XGP typically presents with an already non-functioning kidney, the radiological and pathological endpoint of long-standing, unrelieved obstruction, while EPN illustrates how rapidly that same continuum can compress into a fulminant, life-threatening presentation.

Because these entities overlap clinically and are sometimes used inconsistently across the literature, the practical, working distinction we adopt throughout this review—consistent with the imaging-based literature reviewed in Section 9—is that pyonephrosis is present whenever an obstructed collecting system contains frankly purulent fluid (confirmed by needle aspiration, at the time of drainage, or at surgery), whereas “hydronephrosis” describes the same obstructive anatomy in the absence of infection. Conversely, a purely radiological impression of purulence is treated as suspected rather than confirmed pyonephrosis.

For the purpose of weighing evidence, every clinical source cited in this review was additionally assigned to one of five population categories, and each conclusion is restricted to the category in which it was generated: (A) confirmed pyonephrosis—frank pus documented at aspiration, drainage, or surgery, with or without a positive collecting-system culture; (B) infected hydronephrosis—a dilated, obstructed system with culture-positive or clinically infected urine but no requirement for gross purulence; (C) obstructive pyelonephritis—obstruction, usually by a stone, with clinical or laboratory signs of infection but without sampling of the collecting-system contents as an entry criterion; (D) stone-associated sepsis or infected urolithiasis identified from administrative diagnosis codes; and (E) upper-tract obstruction enrolled irrespective of infection, cited only for technical outcomes. Categories B–D contain an unknown proportion of patients with true pyonephrosis, and category E a smaller one still, so evidence from them is indirect for pyonephrosis proper. The category of each key source is stated where the source is discussed.

## 5. Epidemiology and Risk Factors

Pyonephrosis is uncommon relative to the broader burden of urinary tract infection and urolithiasis, but it is far from rare among patients presenting with obstructive pyelonephritis, and it belongs squarely within the spectrum of kidney diseases responsible for AKI. In a cross-sectional study of 550 adult patients admitted with pyelonephritis to a tertiary-care naval hospital in India (convenience sample), the prevalence of pyonephrosis was 10.9% (60/550; 95% CI 8.3–13.5%), with a mean patient age of 54.6 ± 12.1 years and a male predominance (68.3%) [26]; pyonephrosis was defined radiologically, with drainage of cloudy urine or pus at decompression (frank pus in 12 of the 60 cases), and, because the denominator was a referral population admitted with pyelonephritis, the figure cannot be read as a population prevalence. *Escherichia coli* was the most common causative organism (33.3%), followed by Pseudomonas species (15%), and flank pain with or without fever was the most frequent presenting symptom (76.7%) [26]. Among patients specifically presenting with upper urinary tract calculi, calculous obstructive pyonephrosis has been estimated—in a figure quoted by Lu et al. from earlier literature—to complicate approximately 3.2% of cases [10], whereas nephrolithiasis is the single most common underlying cause of pyonephrosis in adults, reflecting the central role of stone-related obstruction discussed in Section 6.

Beyond urolithiasis, recognized risk factors and associations include diabetes mellitus, pregnancy, immunosuppression (including solid-organ transplantation), congenital ureteropelvic junction or ureteral anomalies, neurogenic bladder and other causes of functional obstruction, prior ipsilateral urological instrumentation or surgery, and malignant ureteral obstruction, most often from urothelial carcinoma or extrinsic pelvic malignancy in older patients. Among patients with renal calculus disease specifically, a retrospective comparison of 91 patients with pyonephrosis and 410 without (n = 501) identified longer duration of symptoms, staghorn calculi, moderate-to-severe hydronephrosis, associated ipsilateral ureteropelvic junction obstruction or ureteric calculus, multiple stones, and a non-functioning kidney as predictors of pyonephrosis, with a history of prior urological surgery emerging additionally in multivariate analysis [27]—a profile that essentially describes long-standing, high-grade, recurrent obstruction, consistent with pyonephrosis, often representing the end-stage of chronic rather than only acute obstructive processes. A non-functioning kidney was present in 71.4% of the pyonephrosis group compared with 1.7% of the comparison group [27], but this cross-sectional association cannot establish whether loss of function preceded or followed the suppurative episode, and it does not by itself demonstrate a relationship with subsequent CKD.

Population-level data from the United States Nationwide Inpatient Sample (1999–2009, a weighted estimate of 396,385 adults hospitalized with infected urolithiasis identified from diagnosis codes) documented that the incidence of hospitalization with infected urolithiasis was approximately twice as high in women as in men (rising from 15.5 to 27.6 per 100,000 women and from 7.8 to 12.1 per 100,000 men), that the proportions with associated sepsis (6.9% to 8.5%) and severe sepsis (1.7% to 3.2%) rose over the study period even as in-hospital mortality remained stable (0.25% to 0.20%), and that the use of percutaneous nephrostomy among patients undergoing immediate decompression declined from 16.1% to 11.2% over the same interval [28]—trends that the original authors attributed in part to evolving practice patterns and the increasing use of retrograde ureteral stenting, a comparison we return to in Section 10.

The female predominance in this dataset and the male predominance in the tertiary series cited above [26] are not contradictory, because the two figures answer different questions. The Nationwide Inpatient Sample figure is a population incidence of hospitalization for any coded infection accompanying urolithiasis (category D), a broad category that includes cystitis and unspecified urinary tract infection, as well as pyelonephritis; the authors themselves noted that the excess in women may simply reflect the higher prevalence of urinary tract infection in women [28]. The tertiary series, by contrast, reports the sex mix of patients with pyonephrosis within a single referral cohort admitted with pyelonephritis to a naval hospital—where a male-skewed catchment population is plausible—and the stone disease that underlies most pyonephrosis is itself more frequent in men. Other series confirm that the sex ratio depends on the cohort: women accounted for 56.5% of patients with pyonephrosis, compared with 38.2% of those with uninfected hydronephrosis, in an Italian cohort in which female sex was associated with pyonephrosis on univariable (odds ratio 2.34, 95% CI 1.12–4.78) but not on multivariable analysis [6], whereas men predominated (181 of 315, 57.5%) in a multicenter United States series of percutaneous nephrostomies for pyonephrosis or infected hydronephrosis [29]. Female sex is therefore best regarded as a risk factor for infection complicating urolithiasis at the population level, but not as an established independent predictor of pyonephrosis among patients who already have an obstructed system.

## 6. Etiology and Pathophysiology

### 6.1. The Obstruction–Infection Axis

The pathophysiology of pyonephrosis follows a single, well-characterized final common pathway: mechanical obstruction of the urinary tract—most commonly by a ureteral or renal calculus, but also by ureteropelvic junction obstruction, ureteral stricture (ischemic, iatrogenic, or post-inflammatory), extrinsic compression, or intraluminal tumor—produces urinary stasis and progressive pelvicalyceal dilatation. Superimposed bacterial infection, reaching the stagnant urine by the ascending transurethral route in the great majority of cases and occasionally by hematogenous seeding, then converts sterile hydronephrosis into an infected, closed-space collection. Because the collecting system cannot decompress itself, intrarenal pressure rises, medullary and then cortical blood flow is compromised, and the combination of ischemia and bacterial toxins drives progressive suppurative necrosis of the renal parenchyma—the histological correlate of the pus that is aspirated at percutaneous drainage or found at nephrectomy, and the plausible anatomical substrate of a transition from reversible AKI to irreversible nephron loss. This sequence explains two clinically pivotal features of the disease: first, that source control (relief of obstruction) is at least as important as antimicrobial therapy in halting progression, because antibiotics alone cannot sterilize a closed, pressurized, poorly perfused space; and second, that the urine sampled from the bladder or ureter distal to the obstruction may be entirely sterile even when the obstructed renal pelvis itself is grossly purulent, since bladder or midstream urine culture is frequently negative or discordant when the obstructed renal pelvis is infected [10,11,12,13]—a discordance that has direct implications for microbiological sampling, as discussed in Section 7.

The consequences of this sequence extend beyond the acute episode. Urinary tract obstruction is among the best-characterized experimental models of progressive renal scarring: sustained obstruction produces tubular dilatation and cell death by apoptosis and necrosis, recruitment of interstitial macrophages, and myofibroblast-driven deposition of extracellular matrix, culminating in tubulointerstitial fibrosis and permanent nephron loss [30,31]. Superimposed suppuration plausibly accelerates each of these steps. This offers a plausible—but so far untested—mechanistic bridge between an episode of pyonephrosis and the acute kidney injury-to-chronic kidney disease continuum now recognized in nephrology, in which an apparently discrete acute insult leaves a residue of reduced functional reserve and predisposes to subsequent decline [32,33]. These data derive from experimental models of sterile obstruction and from AKI of other causes; no longitudinal study has yet measured the incidence of CKD after pyonephrosis, and the link should therefore be regarded as a hypothesis. It may also explain why recovery after decompression is better conceived as a spectrum than as a binary outcome, and why the interval between obstruction and drainage matters not only for survival but for the nephron mass that survives with the patient.

**Figure 1:**
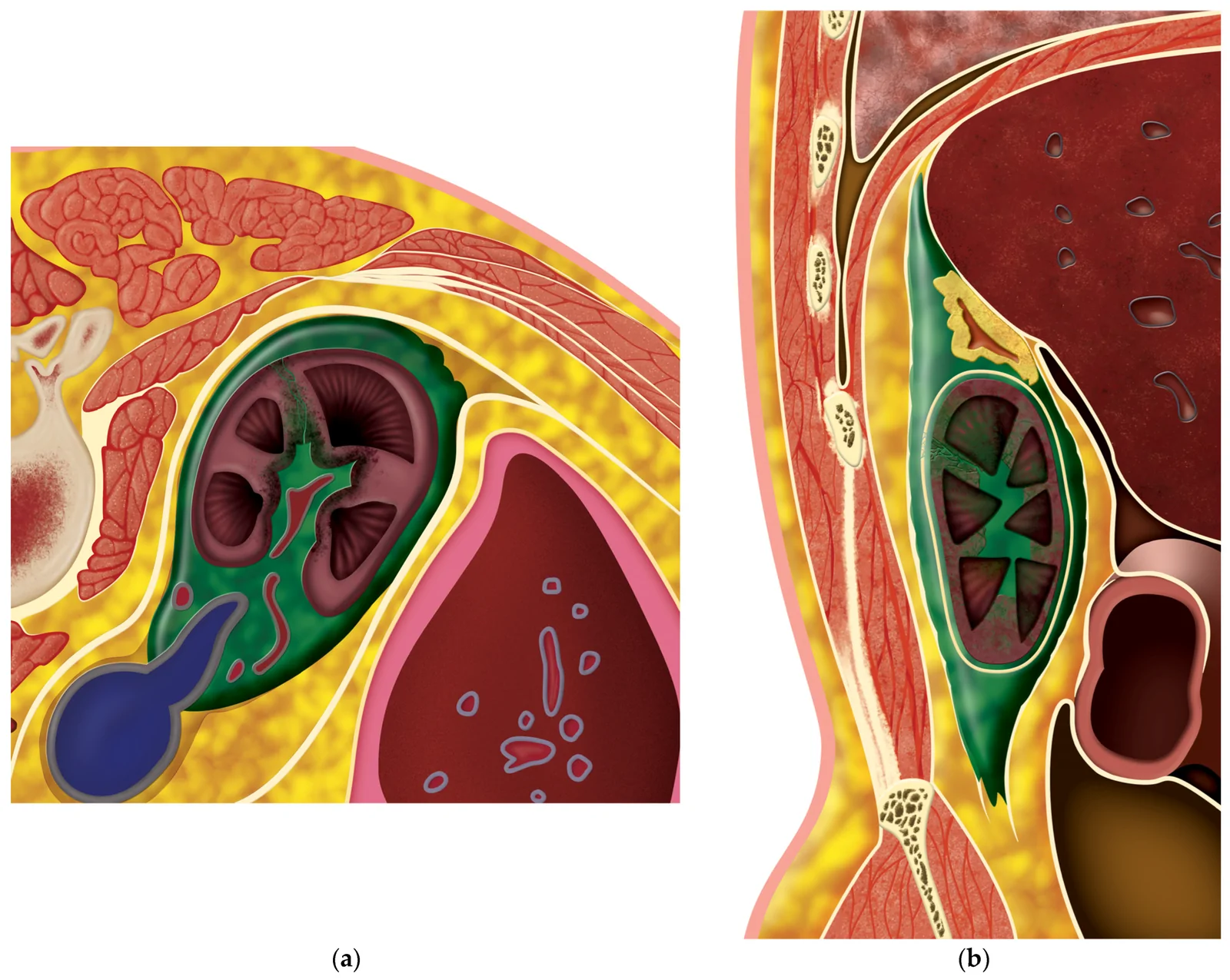
Illustrated sections through a pyonephrotic kidney, showing the pattern of purulent dissemination once pus has breached the renal cortex and begun to spread into the perinephric space: (**a**) Axial section; (**b**) Sagittal section. Original illustrations created by the authors in collaboration with medical illustrator Marius Filip.

Once the process described above has proceeded far enough, pus and inflammatory debris breach the renal capsule itself and extend into the perinephric space, as shown in Figure 1. This transcapsular extension marks a clinically important threshold: it signals that parenchymal destruction is no longer confined to the collecting-system margin, that the substrate for psoas and perinephric abscess or fistula formation is now present, and that renal salvage becomes correspondingly less likely the longer decompression is delayed.

### 6.2. Xanthogranulomatous Pyelonephritis

Xanthogranulomatous pyelonephritis is a rare (representing well under 1% of chronic pyelonephritis), chronic, destructive variant of the same obstruction–infection axis, in which normal renal parenchyma is progressively replaced by granulomatous inflammatory tissue rich in lipid-laden (“foamy”) macrophages. First described by Schlagenhaufer in 1916 [34,35], XGP was characterized in the landmark 1978 critical analysis of 26 cases by Malek and Elder as a disease of middle-aged or older women, who presented with anemia, chronic febrile illness, a painful, tender flank mass, and recurrent urosepsis; a renal mass was seen urographically in 62% of patients, nephrolithiasis in 38%, and a functionless kidney in 27%, and the correct preoperative diagnosis was made in only a single case, reflecting the condition’s long-recognized capacity to mimic renal malignancy [36]. Obstructing calculi, often staghorn in configuration, are present in the substantial majority of cases. Two complementary classifications are in use. Malek and Elder staged the disease by anatomical extension into Stage I (nephric: confined to the renal parenchyma), Stage II (perinephric: extension into the perinephric fat), and Stage III (paranephric: extension into the pararenal space and adjacent retroperitoneum) [36], whereas a separate morphological classification distinguishes the diffuse form (involvement of the entire kidney, the most common and most surgically challenging pattern) from the less common segmental and focal forms [35]. The distinction matters practically because focal or segmental disease can occasionally be managed with antibiotics or partial nephrectomy, whereas diffuse disease essentially always requires total nephrectomy, which was the treatment used in the majority (21/26) of the Malek–Elder series [35,36].

### 6.3. Emphysematous Pyelonephritis

Emphysematous pyelonephritis is a necrotizing, gas-forming infection that shares the obstructive-suppurative mechanism of pyonephrosis but is radiologically distinguished by gas within the renal parenchyma, collecting system, or perinephric tissues, produced by gas-forming organisms (predominantly *Escherichia coli*) fermenting tissue glucose under conditions of poor tissue perfusion. It occurs overwhelmingly in patients with diabetes mellitus—itself among the leading causes of CKD worldwide—and carries a historically high mortality. The clinico-radiological classification proposed by Huang and Tseng in 2000 remains the most widely used framework for staging and treatment planning: Class 1 disease is limited to gas within the collecting system only; Class 2 involves gas within the renal parenchyma without extension beyond the kidney; Class 3 is subdivided into 3A (extension of gas or abscess into the perinephric space) and 3B (extension into the pararenal space); and Class 4 denotes bilateral emphysematous pyelonephritis or disease in a solitary functioning kidney [37]. This classification correlates with prognosis and guides management: Classes 1 and 2 generally respond well to antibiotics with or without percutaneous drainage; Class 3 or 4 disease with fewer than two of the four adverse risk factors identified in that series (thrombocytopenia, acute renal function impairment, disturbance of consciousness, and shock—diabetes itself is not a discriminating factor, being present in 96% of the cohort) can usually still be managed with antibiotics plus percutaneous drainage, with an 85% success rate, whereas the same strategy failed in 92% of patients with two or more risk factors (versus 15% of those with fewer), who therefore more often require nephrectomy; Class 4 (bilateral or solitary-kidney) disease is managed within the same risk-stratified framework [37].

## 7. Microbiology

The microbiology of pyonephrosis mirrors that of complicated upper urinary tract infection more broadly. Gram-negative *Enterobacterales* predominate, with *Escherichia coli* the single most commonly isolated organism in most series [26], followed variably by *Proteus mirabilis*, *Klebsiella pneumoniae*, *Pseudomonas aeruginosa* [26], and *Enterococcus* species among Gram-positive isolates. Urease-producing organisms—chiefly *Proteus*, but also some strains of *Klebsiella* and *Pseudomonas*—hydrolyze urea to ammonia, alkalinizing the urine and promoting precipitation of struvite (magnesium ammonium phosphate) and carbonate apatite, the so-called infection stones that frequently form large, branching staghorn calculi; these stones both cause the obstruction that initiates pyonephrosis and physically harbor bacteria within their matrix, making them very difficult to sterilize without their removal. Gas-forming organisms, again predominantly *E. coli*, underlie emphysematous pyelonephritis (Section 6.3). Fungal pyonephrosis, most often due to *Candida* species and occasionally to filamentous fungi, occurs principally in instrumented, diabetic, or immunocompromised patients and in those with long-term indwelling urinary devices. Tuberculous pyonephrosis represents an end-stage manifestation of genitourinary tuberculosis, in which chronic caseating destruction can produce a non-functioning, calcified “putty” or “cement” kidney; its recognition requires a specific index of suspicion, acid-fast staining, and mycobacterial culture, since routine bacterial cultures will be uninformative.

A microbiological feature of direct clinical importance is the frequent discordance between bladder or midstream urine culture and the true infective status of the obstructed kidney: because the obstructing lesion sequesters the infected renal-pelvis contents from the bladder, a negative midstream culture does not exclude infection above the obstruction [10]. The primary data are small and heterogeneous in population but consistent in direction. In 23 patients with confirmed pyonephrosis treated by percutaneous nephrostomy, renal urine cultures were positive in 16 of 21 and provided bacteriological information not available from bladder urine cultures in 11 [11]; in a prospective study of 73 patients with obstructing ureteral stones undergoing ureteroscopy, pelvic urine was culture-positive in 58.9% but midstream urine in only 28.8%, giving midstream culture a sensitivity of 30.2% for infected pelvic urine [12]; in a contemporary cohort of obstructed, infected ureterolithiasis, pathogens isolated from the renal pelvis were absent from the midstream specimen in 52 of 168 patients (31%) [13]; and a multicenter series of 315 percutaneous nephrostomies for pyonephrosis or infected hydronephrosis likewise noted clinically important disparities between nephrostomy and bladder cultures [29]. Across these series, bladder or midstream culture failed to identify the upper-tract pathogen in roughly one-third to two-thirds of patients—a range, derived from small samples without confidence intervals, that is more defensible than any single summary proportion. Conversely, when pyonephrosis was defined by visibly purulent urine at drainage, collecting-system urine was culture-positive in 86.9% of pyonephrotic versus 14.4% of hydronephrotic systems [6]. This discordance is the principal reason that international guidelines specifically recommend obtaining a culture from the collecting system itself—via the nephrostomy or at the time of stent placement—rather than relying on bladder urine alone [38,39,40]. Rising rates of extended-spectrum beta-lactamase (ESBL)-producing and other multidrug-resistant *Enterobacterales* among community and hospital uropathogens further complicate empirical antimicrobial selection, reinforcing the importance of local antibiogram data and of prompt de-escalation once culture and susceptibility results—from both pre- and post-decompression specimens—become available (see Section 10.3).

## 8. Clinical Presentation and Laboratory Findings

The classic presentation of pyonephrosis combines flank pain, fever with chills or rigors, and pyuria, sometimes accompanied by a palpable flank mass and, in more advanced cases, overt septic shock [26]. In a recent cross-sectional series, flank pain with or without fever was the presenting complaint in more than three-quarters of patients [26]. However, the clinical spectrum is broad, ranging from an indolent picture of low-grade fever, malaise, and dull flank discomfort—more typical of chronic, long-standing obstruction, such as that underlying xanthogranulomatous pyelonephritis—to fulminant urosepsis evolving over hours. Presentation can be notably atypical or blunted in diabetic and other immunocompromised patients, in whom fever and localizing pain may be attenuated even as the underlying disease is advanced; this is a recognized diagnostic trap, since a reassuring absence of fever should not be taken as evidence against pyonephrosis in a patient with obstruction and other signs of infection.

Laboratory findings typically include leukocytosis (though leukopenia may be seen in severe sepsis), elevated inflammatory markers such as C-reactive protein and procalcitonin, pyuria and bacteriuria on urinalysis when the obstruction is incomplete enough to allow urine to reach the bladder, and variable derangement of renal function, which is most pronounced in bilateral disease or obstruction of a solitary or transplanted kidney—an acute-on-chronic pattern that should prompt the same systematic search for a reversible, obstructive cause that any nephrologist applies to unexplained AKI. Blood and urine cultures should be obtained before antimicrobial therapy whenever feasible, recognizing the important caveat, discussed above, that a sterile bladder urine culture does not exclude pyonephrosis [10,11,12,13]. Systemic inflammatory response syndrome (SIRS) criteria and established early-warning scores remain useful bedside tools for recognizing evolving sepsis and triaging the urgency of decompression; current international sepsis guidance, however, advises against relying on the quick Sequential Organ Failure Assessment (qSOFA) alone for screening, given its low sensitivity, while noting that a positive qSOFA should still alert the clinician to possible sepsis and retains value for outcome prediction once sepsis is established (see Section 10.1). Elevated procalcitonin in particular has been highlighted as a biomarker that can help prioritize ultra-early drainage in the sickest patients [41].

## 9. Morphological Diagnosis: Imaging

### 9.1. Ultrasonography

Ultrasonography is the appropriate first-line investigation in suspected pyonephrosis: it is rapid, avoids ionizing radiation, requires no intravenous contrast, and can be performed at the bedside in critically ill patients. The defining sonographic feature is a dilated pelvicalyceal system containing echogenic material, in contrast to the anechoic fluid of simple hydronephrosis. Additional described features include dependent, low-level internal echoes that layer or shift with changes in patient position, distinct fluid–debris or fluid–fluid levels, diffuse or focal thickening of the urothelium (a wall thickness exceeding roughly 2 mm is considered abnormal, though this finding is nonspecific) [42], and, when gas-forming organisms are present, hyperechoic foci with “dirty” posterior shadowing.

**Figure 2:**
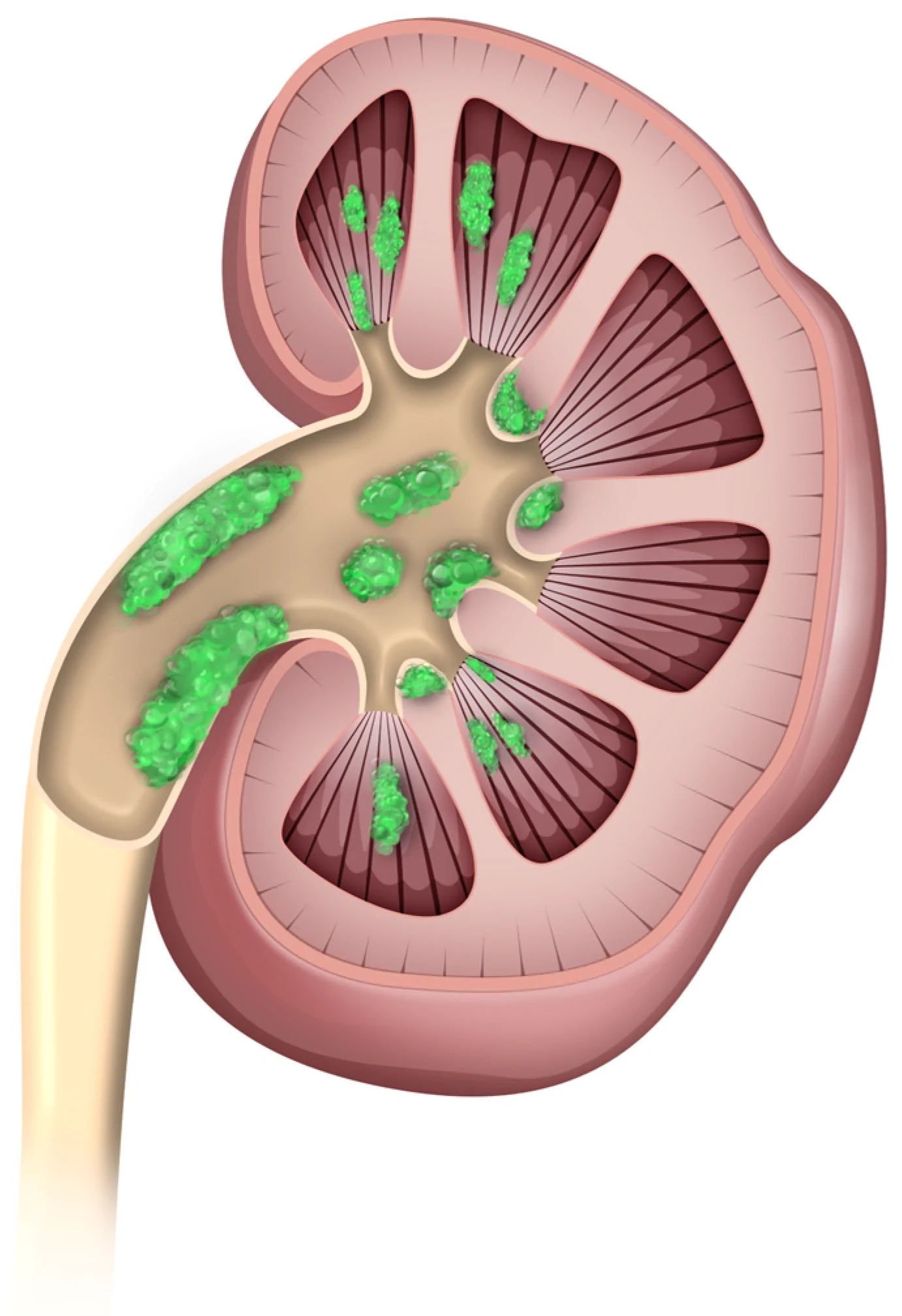
Illustrated coronal section through an early pyonephrosis case, showing markedly inhomogeneous distribution of purulent debris within the dilated collecting system—a recognized confounding factor for morphological diagnosis on imaging, since the region sampled can determine whether the study appears frankly infected or deceptively bland. Original illustration created by the authors in collaboration with medical illustrator Marius Filip.

The foundational prospective evaluation of these signs, published in 1983, reviewed 92 hydronephrotic kidneys in 73 patients and found that, among 34 patients with clinical suspicion of renal infection, internal echoes within the dilated collecting system identified pyonephrosis with 90% sensitivity and 97% specificity, while the complete absence of internal echoes in patients without clinical evidence of infection carried 100% specificity for sterile hydronephrosis [43]. A subsequent re-evaluation of the same sonographic criteria in 1985, however, found a substantially lower sensitivity of 62% (with specificity maintained at 100%) [44], a discrepancy that has never been fully reconciled and that should temper unqualified citation of the original, more favorable figures: a normal-appearing, anechoic collecting system on ultrasound reduces but does not reliably exclude the possibility of infection, particularly in early or low-grade disease. Both were small, single-center series from the early era of gray-scale sonography, neither reported confidence intervals, and the sensitivity of sonography for confirmed pyonephrosis therefore remains uncertain within the range that these two estimates define. Figure 2 illustrates this pitfall schematically: a coronal section through an early pyonephrosis shows markedly inhomogeneous distribution of purulent debris within the dilated collecting system, such that the appearance—and the apparent risk of infection—can differ substantially from one part of the collecting system to another, a recognized confounding factor for morphological diagnosis that is easy to underestimate from textbook, single-image descriptions. Practical limitations of ultrasonography include reduced sensitivity in obese patients, difficulty characterizing retroperitoneal or perinephric extension of infection, and gas artifact, which can both signal emphysematous infection and, paradoxically, obscure other collecting-system detail. Despite these limitations, point-of-care ultrasound performed by the treating clinician has an established and growing role in rapid triage, particularly in resource-limited or critical-care settings where immediate cross-sectional imaging is not available [45].

### 9.2. Computed Tomography and Hounsfield-Unit Quantification

CT is the principal modality for staging pyonephrosis, characterizing its underlying cause, and detecting extrarenal complications, and non-contrast acquisition alone is frequently sufficient for these purposes, given that the affected kidney is often poorly or non-functioning and therefore excretes little or no contrast. In the foundational comparative CT series—17 pyonephrotic and 20 uninfected hydronephrotic kidneys, all subsequently confirmed by percutaneous nephrostomy—pyonephrosis was more often associated with increased pelvic wall thickness and with more severe perirenal fat stranding than uninfected hydronephrosis, but the authors were careful to note that, for any individual patient, these morphological findings were often not diagnostic in isolation, and that the presence of clinical signs of infection alongside hydronephrosis on CT was in fact a more sensitive indicator of pyonephrosis than the CT findings themselves [46]. This important caveat—that CT morphology alone frequently under-discriminates pyonephrosis from bland hydronephrosis—is precisely what has motivated the search for objective, quantitative CT adjuncts described below. Additional morphological features include pelvicalyceal dilatation, renal enlargement, an accentuated “striated” nephrogram more pronounced than that seen with bland obstruction, and, in a minority of cases, frank gas within the collecting system or parenchyma, which, in a patient without recent instrumentation, is highly specific for infection [47]. Contrast-enhanced acquisition, where renal function permits, further defines the underlying obstructing lesion and the degree of preserved parenchymal enhancement.

Because most of these signs are indirect, quantitative attenuation (Hounsfield unit, HU) measurement of the fluid within the dilated collecting system has been increasingly proposed as an objective adjunct that can be obtained from the same non-contrast CT already performed for stone protocol, without additional radiation or contrast exposure. Purulent fluid, laden with cells and proteinaceous debris, attenuates more than clear urine, and several cohorts have reported higher mean HU values in pyonephrosis than in hydronephrosis [6,10,48]. All of these studies, however, were single-center and retrospective in design; each derived its threshold by receiver operating characteristic analysis and tested it in the same sample; none has been externally validated, and they differ in population, reference standard, and measurement protocol; the thresholds reported below are therefore study-specific findings rather than transferable cut-offs. In a single-center cohort of 122 patients undergoing nephrostomy or ureteral catheter placement for obstructive uropathy of mixed etiology (stones in 79.5%), pyonephrosis was defined by the operator’s visual impression of cloudy or purulent urine at drainage, without laboratory confirmation (46 pyonephrosis, 76 hydronephrosis), and attenuation was measured on 3 mm non-contrast reconstructions by two blinded radiologists (interobserver intraclass correlation coefficient 0.90, 95% CI 0.85–0.93); a threshold of 6.3 HU in the region of interest discriminated pyonephrosis from hydronephrosis with 71.6% sensitivity and 71.5% specificity (AUC 0.76, 95% CI 0.66–0.85), and a slightly higher threshold of 7.3 HU was associated with post-drainage sepsis, with 76.5% sensitivity and 74.3% specificity (AUC 0.79, 95% CI 0.71–0.90) [6]. In a separate retrospective cohort of 159 patients (214 individual kidneys), in which collecting-system fluid was classified by percutaneous needle aspiration and culture of the aspirate, mean HU values were markedly higher in pyonephrotic (10.97 ± 6.68 HU) than in hydronephrotic kidneys (2.30 ± 5.02 HU, *p* < 0.001), and a threshold of 8.46 HU yielded 68.4% sensitivity and 92.6% specificity, albeit without confidence intervals [48]. A third series, restricted to 240 patients with upper urinary tract stones, reported mean HU of 15.46 in pyonephrosis versus 5.5 in hydronephrosis, with a best threshold of 9.5 HU (sensitivity 71.4%, specificity 70.2%) but an AUC of only 0.613—lower than that of leukocyte count or C-reactive protein in the same cohort [10]. An earlier study of 105 patients with nephrostomy, in which pyonephrosis was defined by clinical criteria, proposed a threshold of 9.21 HU (sensitivity 65.9%, specificity 87.9%) [49], whereas a study that used renal pelvic urine culture as the reference standard found the opposite relationship, with culture-positive systems showing lower attenuation and a best threshold of 0 HU [50]. Proposed thresholds thus range from 0 to 9.5 HU, and AUCs, where reported, from 0.61 to 0.79. This heterogeneity across studies partly reflects sampling technique: because purulent debris tends to layer dependently rather than distribute evenly through the dilated pelvis, a single-region-of-interest measurement is sensitive to exactly where in the collecting system the sample is drawn—the same phenomenon illustrated by Figure 2 above—whereas sampling several representative areas of the dilated system, including its dependent portion, captures the heterogeneity of the fluid more reliably: in one series of 181 patients with calculous hydronephrosis (71 with pyonephrosis), in which a single-slice measurement achieved an AUC of only 0.633, the range of mean HU values across different slices of the hydronephrotic region discriminated pyonephrosis with 88.7% sensitivity and 86.4% specificity, compared with 50.7% and 70.9% for a single-slice measurement [51]. In practice, no HU threshold has been externally validated, and clinicians should treat an elevated or heterogeneous attenuation value as one supportive element within the overall clinical and sonographic picture rather than as a stand-alone diagnostic cut-off.

### 9.3. Magnetic Resonance Imaging and Diffusion-Weighted Imaging

MRI is not required for the diagnosis of most cases of pyonephrosis but may offer additional non-invasive discrimination from sterile hydronephrosis where CT and ultrasound are equivocal, where iodinated contrast or ionizing radiation is specifically to be avoided (for example, in pregnancy or in patients with pre-existing CKD and limited renal reserve), or where renal function is markedly impaired. On conventional T1- and T2-weighted sequences, hydronephrosis and pyonephrosis can appear similar, since both are hypointense on T1 and hyperintense on T2 unless the dependent collecting-system contents are sufficiently proteinaceous or cellular to alter signal. Diffusion-weighted imaging (DWI) can help to resolve this ambiguity: because the densely cellular, viscous character of pus markedly restricts water diffusion, the pyonephrotic renal pelvis is strikingly hyperintense on high-b-value DWI and correspondingly hypointense (i.e., shows a markedly reduced apparent diffusion coefficient, ADC) on the derived ADC map, whereas the free water of simple hydronephrosis diffuses much more readily and shows a correspondingly high ADC. In an early description of this technique, applied to 12 patients (four with pyonephrosis, eight with hydronephrosis confirmed microbiologically), the mean ADC of the pyonephrotic renal pelvis was 0.64 ± 0.35 ×10^−3^ mm^2^/s, compared with 2.98 ± 0.65 ×10^−3^ mm^2^/s for the hydronephrotic renal pelvis [52]—a difference of roughly fourfold that the authors concluded made DWI-MRI a reliable tool for this distinction. However, with only four pyonephrotic kidneys, no ADC threshold, accuracy estimate, or confidence interval could be derived, and the finding still awaits confirmation in larger, prospective series. Moreover, measured ADC values depend on the b-values, field strength, scanner, sequence, and region-of-interest placement used and vary between observers, so these figures describe that study’s protocol and patients and cannot be transferred to other settings as a diagnostic cut-off; the qualitative observation of markedly restricted diffusion relative to simple hydronephrosis is the more generalizable finding.

### 9.4. Nuclear Medicine and Functional Renal Assessment

Radionuclide studies play a limited role in the acute diagnosis of pyonephrosis but an important one in subsequent management planning, and are the most direct bridge in this review to the biomarker- and function-based assessment that underpins CKD staging more broadly. Technetium-99m dimercaptosuccinic acid (DMSA) scintigraphy assesses cortical uptake and scarring, while technetium-99m mercaptoacetyltriglycine (MAG3) renography assesses tubular function and drainage; both are used to quantify split (differential) renal function and to help decide, once the acute infection has been controlled, whether the affected kidney retains sufficient function to justify reconstructive treatment of the underlying obstruction or whether it should instead be considered for nephrectomy (see Section 10.5). Because active obstruction and infection themselves depress apparent renal function, these studies are most informative when performed after a period of temporary decompression (typically by nephrostomy) rather than during the acute presentation, allowing reversible, infection- and pressure-related functional impairment to be distinguished from the truly irreversible loss of function that characterizes an end-stage, destroyed kidney.

Alongside these functional studies, tubular injury biomarkers have been explored as a means of quantifying obstructive renal damage and tracking its reversibility. Urinary kidney injury molecule-1 (KIM-1) and neutrophil gelatinase-associated lipocalin (NGAL) rise with the severity of congenital obstruction [53], and urinary NGAL together with serum cystatin C distinguish infants with surgically managed ureteropelvic junction obstruction from healthy controls, falling significantly after relief of obstruction [54]. In adults, urinary KIM-1 decreases significantly following treatment of hydronephrosis and is higher in obstructed than in non-obstructing stone disease, whereas NGAL is confounded by leukocyturia—an important caveat in precisely the infected, obstructed population considered here [55]. These remain small, largely hypothesis-generating studies, and no biomarker is yet validated for the salvage-versus-nephrectomy decision in pyonephrosis; they nonetheless indicate a plausible route toward objective, early quantification of the reversible component of obstructive renal injury.

### 9.5. Extension Beyond the Kidney, and Historical and Adjunct Techniques

**Figure 3:**
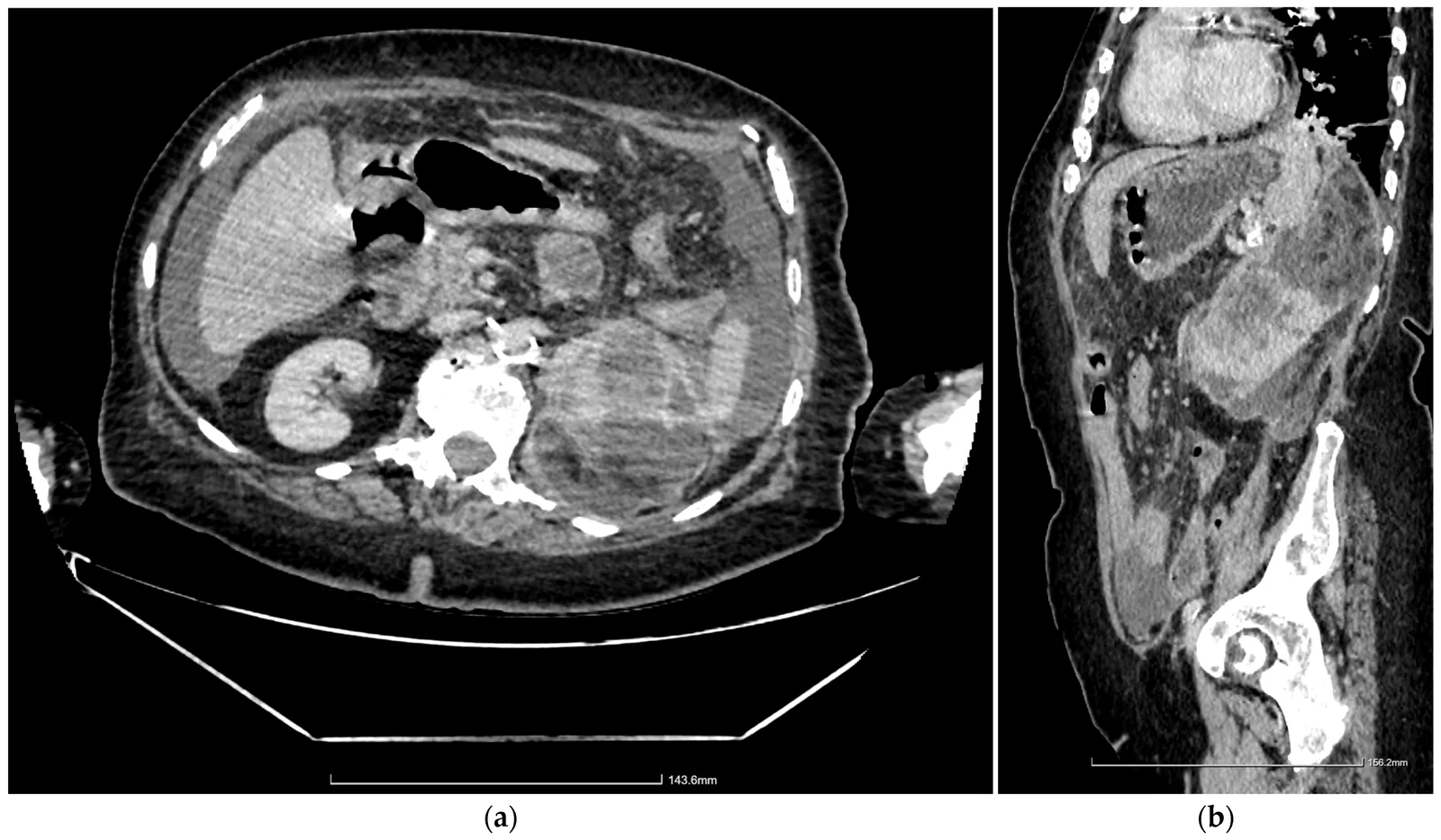
Contrast-enhanced CT images from a 74-year-old woman with advanced calculous pyonephrosis that had perforated into the peritoneal cavity, confirmed intraoperatively by frank pseudomembranous peritonitis: (**a**) Axial section; (**b**) Sagittal section. The images are anonymized, and written informed consent was obtained from the patient.

Figure 3 illustrates the far end of this morphological spectrum. Untreated or inadequately drained pyonephrosis can progress to spontaneous rupture with intraperitoneal or retroperitoneal extension of infected material, an uncommon but life-threatening complication. The images show a 74-year-old woman under our care with advanced calculous pyonephrosis that had perforated into the peritoneal cavity, an event confirmed intraoperatively by frank pseudomembranous peritonitis; the axial (Figure 3a) and sagittal (Figure 3b) contrast-enhanced CT sections obtained before surgery demonstrate how far the morphological appearance of pyonephrosis can evolve once containment by the renal capsule and collecting system has failed entirely, and underline why the threshold for suspecting the diagnosis—and for triggering urgent decompression—should be set low rather than reserved for cases with unambiguous imaging.

Plain abdominal radiography and intravenous urography (IVU/IVP), once the mainstay of investigation, are now largely of historical interest in this context: a fragmented staghorn calculus on plain film was described as a specific, if uncommon, radiological sign of underlying pyonephrosis [56], and IVU classically demonstrated a poorly or non-functioning obstructed kidney, but both techniques have been superseded by the speed, sensitivity, and functional information provided by ultrasound and CT and are seldom used today to establish this diagnosis.

## 10. Contemporary Clinical Management

### 10.1. Initial Resuscitation and Recognition of Urosepsis

Because pyonephrosis is frequently the anatomical substrate of urosepsis, initial management follows the same principles as sepsis of any origin: prompt recognition using validated bedside criteria, blood and urine cultures obtained before (or without delaying) antimicrobial therapy, intravenous fluid resuscitation, and early broad-spectrum empirical antibiotics, escalating to vasopressor support and intensive care as needed, in line with the Surviving Sepsis Campaign guidelines [57], which recommend structured screening of acutely ill, high-risk patients using SIRS criteria or an established early-warning score rather than qSOFA alone. What distinguishes urosepsis of obstructive origin from most other sepsis sources, however, is that antimicrobial therapy alone cannot achieve source control: the obstructed, pressurized, poorly perfused collecting system must be mechanically decompressed, and this step should not be delayed while awaiting resolution of hemodynamic instability, since delay has been associated with worse outcomes in observational cohorts (see Section 11).

### 10.2. Urgent Decompression: Percutaneous Nephrostomy Versus Retrograde Ureteral Stenting

Urgent decompression of the obstructed, infected collecting system is the cornerstone of pyonephrosis management and is strongly recommended by both European and American urological guidelines [38,39]; the recommendation rests on physiological rationale and observational data rather than on randomized comparison with non-drainage, and the American guideline accordingly grades its supporting evidence as Grade C (Section 11). Two techniques are available: percutaneous nephrostomy (PCN), placed under ultrasound or fluoroscopic guidance directly into the renal collecting system, and retrograde ureteral (double-J) stenting, placed cystoscopically. At the time of emergency PCN placement in a septic patient, diagnostic pyelography and additional manipulation of the collecting system should be minimized, since raising intrapelvic pressure in an infected system can itself precipitate septic shock.

The comparative evidence base, summarized in Table 1, is more extensive than for most questions in emergency urology, yet has not resolved into a clear consensus favoring either technique; no trial has been restricted to confirmed pyonephrosis, and the populations enrolled differ between studies. In the first randomized trial to address this question directly, 42 patients with obstructing ureteral calculi and clinical signs of infection (temperature of 38 °C or above and/or leukocyte count of 17,000/mm^3^ or above)—obstructive pyelonephritis, with grossly purulent urine at drainage in only six—were allocated to PCN or retrograde catheterization; there was no significant difference between groups in time to normalization of temperature (2.3 versus 2.6 days) or white blood cell count (2.0 versus 1.7 days) or in length of hospital stay (4.5 versus 3.2 days). The trial was sized (17 patients per group) to detect a one-day difference, its authors acknowledged that power fell below 80%, no confidence intervals were reported, and there was no equivalence or non-inferiority design, so that, with 21 patients per arm, differences of about a day in either direction could not be excluded. Retrograde catheterization required shorter procedure (32.7 versus 49.2 min) and fluoroscopy time (5.1 versus 7.7 min), while collecting-system urine cultures were positive more often after PCN (13 versus 4 of 21 patients; 61.9% versus 19.1%), the single technical failure occurred in the PCN arm and was rescued by retrograde catheterization, cost analysis favored PCN (retrograde catheterization being twice as costly), and back discomfort during the procedure and the use of parenteral narcotics were greater with PCN (37.2 versus 6.5 on a 100-point scale; 38.1% versus 4.8%); the authors concluded that neither technique was superior in promoting clinical recovery [24]. A second randomized trial of 40 patients—which enrolled stone-induced hydronephrosis requiring diversion and was not restricted to infected systems—reached a different practical conclusion: retrograde stent placement failed in roughly one in five patients—more often in those with high (proximal) ureteral stones and in patients over 60 years of age—who were then rescued by PCN, whereas PCN placement succeeded in all patients; intravenous antibiotics were required for more than five days in none of the PCN group but in the majority of the stent group (a difference that did not reach statistical significance), and quality of life improved after PCN while deteriorating after stenting, leading the authors to favor PCN, particularly in febrile, male, and younger patients [25].

Two later randomized trials in infected, obstructed systems gave divergent results. Among 65 analyzed patients with upper urinary tract stones and urosepsis, time to normalization of temperature was shorter after PCN than after stenting (3.0 versus 5.0 days, as extracted in a subsequent meta-analysis [58]) and postoperative C-reactive protein was lower (*p* < 0.05), although seven patients in whom retrograde stenting failed had been excluded from the analysis; the authors concluded that PCN was preferable, particularly in patients with severe inflammation and fever [59]. In 150 patients with ureteral stones and systemic inflammatory response syndrome (SIRS), by contrast, PCN (n = 78) and stenting (n = 72) did not differ significantly in the incidence of post-decompression urosepsis (15.4% versus 22.2%; *p* = 0.283), time to normalization of vital signs (2.35 versus 2.51 days; *p* = 0.072), or length of stay (7.92 versus 8.37 days; *p* = 0.215); purulent fluid at drainage and a higher procalcitonin level were associated with progression to urosepsis after decompression, which identifies pyonephrosis as a marker of risk within this population [60].

Systematic reviews and meta-analyses have not detected statistically significant differences between the two techniques on most pooled clinical endpoints, but their estimates are imprecise and heterogeneous, and they are dominated by non-randomized studies. A 2024 meta-analysis of eight studies of severe urinary tract infection with obstructive urolithiasis—three randomized trials [24,59,65] and five retrospective cohorts—reported pooled mean differences (PCN minus stent) of −0.74 days in time to normalization of temperature (95% CI −1.69 to 0.21; I^2^ = 96%), 0.46 days in time to normalization of white cell count (95% CI −0.30 to 1.21; I^2^ = 85%), and 0.45 days in length of hospital stay (95% CI −2.78 to 3.68; I^2^ = 96%), with an odds ratio of 0.86 for procedural success with PCN (95% CI 0.34–2.20), and concluded that the choice between techniques depends chiefly on urologist or patient preference [58]. These intervals are compatible with no difference, but also with differences of more than a day (in favor of PCN for defervescence, of stenting for leukocyte normalization, and of either technique for length of stay) and with a more than twofold difference in the odds of technical success in either direction; they therefore do not demonstrate equivalence. A separate systematic review and meta-analysis of seven trials (667 patients) with acute upper obstructive uropathy—a population not restricted to infection—similarly found no significant difference in improvement of septic parameters, failure rates, or EQ-5D quality-of-life domains, but noted that PCN was associated with less post-procedural hematuria and dysuria—on which basis its authors favored PCN—at the cost of a longer hospital stay (mean difference 1.82 days, 95% CI 0.79–2.85) [62]. The first trial designed and powered to test non-inferiority, the Dutch STONE trial, randomized 204 patients with obstructing urolithiasis and an indication for drainage (colicky pain in 73%, renal impairment in 62%, and infection in only 35%) and found PCN non-inferior to stenting for time to clinical recovery (1.7 versus 1.5 days; mean difference 0.3 days, 95% CI −0.2 to 0.7, against a prespecified margin of 1 day); crossover because of technical failure was more frequent with PCN (11 of 101 versus 2 of 103 patients), and reintervention within three months was more common after PCN (17% versus 6.4% in the per-protocol analysis) [61]. Because only a minority of participants had infection and none had pyonephrosis confirmed as an entry criterion, this trial supports a similar speed of recovery after either technique in obstructing urolithiasis generally but remains indirect evidence for pyonephrosis.

Set against these randomized and meta-analytic data, large administrative cohorts have reported worse outcomes after PCN than after stenting. In the Nationwide Inpatient Sample (1999–2009), PCN use declined over time and, in propensity-matched analysis, was associated with higher odds of sepsis (odds ratio 1.63, 95% CI 1.52–1.74), severe sepsis (2.28, 95% CI 2.06–2.52), prolonged length of stay (3.18, 95% CI 3.01–3.34), and death (3.14, 95% CI 2.13–4.63), although the propensity score comprised demographic, comorbidity, and hospital variables without any measure of disease severity [28]; in 9172 hospitalizations for sepsis with obstructing ureteral stones (2016–2022), overlap-weighted mortality was 5.5% after PCN versus 2.8% after stenting (odds ratio 2.05, 95% CI 1.31–3.09) [63]. These associations are in all likelihood substantially confounded by indication. In the latter cohort, patients treated with PCN had more often been transferred from another facility (37.4% versus 17.5%), had more comorbidity, and more often required vasopressors and mechanical ventilation, and crude mortality after same-day PCN (6.3%) exceeded that after later PCN (3.1%)—a pattern indicating that the sickest patients are triaged to immediate nephrostomy [63]; in a single-health-system series of 130 patients with obstructive urolithiasis and systemic inflammatory response syndrome, those treated with PCN had larger stones and higher Acute Physiology and Chronic Health Evaluation (APACHE) II scores, and remained more likely to require intensive care (odds ratio 3.23, 95% CI 1.24–8.41) even after adjustment for age, APACHE II score, and comorbidity [64]. Administrative data do not capture hemodynamic status, failed retrograde attempts, or the anatomical reasons for choosing PCN, so residual confounding is probable, and these findings are best regarded as hypothesis-generating rather than as evidence that PCN is inferior—or, conversely, that the two techniques are equivalent.

Reflecting this unresolved uncertainty—rather than proven equivalence—neither the European Association of Urology nor the American Urological Association recommends one modality over the other: both endorse either PCN or retrograde stenting according to local expertise and resources, stone anatomy, and patient factors, with the European guideline issuing a strong recommendation for urgent decompression by either route and describing the two as equally effective, and the American guideline stating that either may be used (a conditional recommendation, for which it grades the evidence as Grade A), while recommending that urine be cultured from the collecting system itself at the time of drainage [38,39]. In practice, most experienced centers favor PCN when stones are large, impacted, or located in the proximal ureter (where retrograde access and stent purchase are least reliable), when retrograde access fails, or when the drained fluid is grossly purulent, and favor retrograde stenting when a percutaneous tube is otherwise undesirable to the patient and retrograde access is technically straightforward; either approach, competently executed, achieves source control in the great majority of patients.

### 10.3. Antimicrobial Therapy

Empirical broad-spectrum antimicrobial therapy—selected according to local resistance patterns, with particular attention to the rising prevalence of extended-spectrum beta-lactamase-producing and other multidrug-resistant *Enterobacterales*—should be initiated promptly, ideally after blood and urine cultures have been obtained but without delaying therapy in a septic patient [40]. Because a sterile standard voiding urine culture does not exclude infection within the obstructed system, a selective urine culture obtained directly from the collecting system at the time of decompression is specifically recommended and, when discordant from bladder voided urine, should guide subsequent antibiotic selection [38,39]. Once culture and susceptibility results are available from both pre- and post-decompression specimens, therapy should be de-escalated to the narrowest effective agent, and duration individualized to clinical response, source-control adequacy, and any residual undrained collection.

### 10.4. Definitive Management of the Underlying Cause

Definitive treatment of the obstructing lesion—stone removal by ureteroscopy, percutaneous nephrolithotomy, or, less often in the acute stone burden typical of pyonephrosis, shockwave lithotripsy; pyeloplasty for ureteropelvic junction obstruction; stricture repair; or oncological management of an obstructing malignancy—should be delayed until sepsis has resolved and infection is controlled, a position reflected in current EAU and AUA guidance [38,39]. A single randomized trial of 107 hemodynamically stable septic patients with impacted ureteral stones found that emergent retrograde ureteroscopic management was feasible, with a shorter hospital stay than PCN (8.2 versus 10.3 days) but greater analgesic consumption [65]; this immediate approach has not displaced the standard, guideline-endorsed strategy of decompression first, followed by interval definitive treatment once the patient has stabilized and infection has cleared.

### 10.5. Nephrectomy: Indications, Timing, and Technique

Nephrectomy is reserved for the kidney that, after adequate decompression and antimicrobial therapy, proves to be irreversibly destroyed and non-functioning, or for the rare case in which sepsis or an undrained abscess persists despite adequate percutaneous or retrograde drainage. Because emergency nephrectomy in the setting of active, unresuscitated sepsis carries substantially higher risk than an elective procedure performed after the acute infection has resolved, nephrectomy is, whenever clinically feasible, deliberately delayed until after initial decompression rather than performed as a primary emergency intervention. Once undertaken, nephrectomy may be performed by an open, laparoscopic (transperitoneal or retroperitoneal), or robotic-assisted approach; the choice depends on surgeon experience, the anatomical extent of disease, and, importantly, the degree of perinephric inflammatory reaction. Both pyonephrosis and its chronic variant xanthogranulomatous pyelonephritis are well-recognized causes of dense, vascularized perinephric adhesions that obliterate normal surgical planes, and a prior episode of pyonephrosis or a diagnosis of xanthogranulomatous pyelonephritis is a recognized risk factor for intraoperative complication and for conversion from a minimally invasive to an open approach: conversion rates of 20–36% have been reported for laparoscopic nephrectomy in xanthogranulomatous pyelonephritis, with dense hilar or perirenal adhesions, a renal length above 12 cm, prolonged time to secure the renal vessels, and right-sided disease predicting conversion in a 66-patient series [66], and open conversion for bleeding was required in one of eight laparoscopic nephrectomies for calculous pyonephrosis in another [67]; surgeons undertaking laparoscopic or robotic nephrectomy in this setting should maintain a correspondingly low threshold for open conversion when dissection proves unsafe.

### 10.6. Assessment of Renal Salvageability

The decision between attempting to salvage the kidney (by decompression followed by definitive reconstructive or ablative stone treatment) and proceeding to nephrectomy should rest, wherever possible, on an objective assessment of renal salvageability rather than on imaging appearance alone, since active infection and obstruction themselves depress apparent function on both cross-sectional imaging and nuclear renography. In practice, this means obtaining split renal function (by MAG3 or DMSA renography, see Section 9.4) after a period of temporary decompression—commonly once nephrostomy output has been established and infection has been brought under control—rather than at initial presentation; meaningful preservation of differential function at this later assessment favors an attempt at renal salvage, whereas a persistently minimal or absent contribution from the affected kidney, particularly when combined with a grossly destroyed parenchymal architecture on imaging, favors proceeding to delayed nephrectomy. This staged assessment is, in essence, the acute-medicine analogue of the differential-diagnosis discipline nephrologists apply to any AKI: distinguishing a reversible component from an established, fixed loss of nephron mass before committing to an irreversible management decision.

### 10.7. Special Populations

Management in pregnancy follows the same core principle of urgent decompression—by retrograde stent or nephrostomy—with the practical modification that fluoroscopy is minimized or avoided in favor of ultrasound guidance, and care is coordinated between urology, obstetrics, and anesthesiology; nephrectomy is only exceptionally required during pregnancy and is deferred, wherever possible, until after delivery. In pediatric patients, the same sequence of urgent percutaneous decompression followed by delayed definitive repair is applied, with laparoscopic or retroperitoneoscopic nephrectomy an option for a destroyed kidney when nephrectomy proves necessary. In patients with a solitary or transplanted kidney, or with pre-existing CKD, or with bilateral disease, the priority shifts still further toward renal preservation: bilateral drainage should be pursued where both kidneys are affected, and nephrectomy is avoided wherever any realistic prospect of functional recovery remains, given the far greater consequence of removing or sacrificing function in a kidney that a patient can least afford to lose.

## 11. Complications and Outcomes

Untreated or inadequately treated pyonephrosis can progress to urosepsis and septic shock, irreversible loss of renal function, spontaneous rupture (intraperitoneal or retroperitoneal, as illustrated in Figure 3) with peritonitis, fistula formation (reno-colic, reno-duodenal, reno-cutaneous), extension to psoas or perinephric abscess, renal vein thrombosis, and progression to xanthogranulomatous pyelonephritis.

The factor most consistently associated with outcome is the timeliness of decompression, although all of the supporting evidence is observational, derives from stone-associated infection or sepsis identified in administrative data rather than from confirmed pyonephrosis, and describes associations rather than causal effects. In a large administrative cohort of 1712 patients with sepsis and ureteral calculi (Nationwide Inpatient Sample, 2007–2009), 78% underwent surgical decompression; mortality was 8.82% among those who were decompressed compared with 19.2% among those who were not (*p* < 0.001), and the absence of surgical decompression remained associated with more than double the odds of death (odds ratio 2.6, 95% CI 1.9–3.7) after adjustment for demographics, comorbidity, and region [8]. Consistent with this, a subsequent systematic review of time-to-decompression in obstructive urosepsis from ureteral calculi, conducted at our institution, concluded that timeliness of drainage—rather than the choice between percutaneous and retrograde technique—was the factor most consistently associated with early outcomes across the available literature, with elevated procalcitonin among the biomarkers best able to flag patients requiring near-immediate decompression [41]; among 311,100 patients admitted with urinary tract infection and a ureteral stone or a renal stone with hydronephrosis (National Inpatient Sample, 2010–2015), decompression two or more days after admission was associated with 29% higher odds of in-hospital death (odds ratio 1.29, 95% CI 1.03–1.63) after adjustment for demographics, comorbidity, and disease severity [9]; and among 9172 hospitalizations for sepsis with obstructing ureteral stones (2016–2022), decompression was associated with lower mortality than conservative management (1.6% versus 4.0%; risk difference −2.4%, 95% CI −3.2 to −1.7) and decompression after more than 24 h with higher odds of death than earlier decompression (odds ratio 2.17, 95% CI 1.38–3.41), although in a landmark analysis the association was no longer significant at 24 h (odds ratio 0.91, 95% CI 0.60–1.40) [63]. After decompression, fever and leukocytosis normalized within two to three days in the randomized trial of obstructive pyelonephritis [24], and renal recovery can be rapid: in an early series of confirmed pyonephrosis treated by percutaneous nephrostomy, azotemia resolved in all 10 azotemic patients, and 25 of 26 patients with long-term follow-up retained function in the previously pyonephrotic kidney [47]. The mortality of approximately 50% sometimes quoted for the combination of obstruction and infection [6] derives from general sepsis cohorts and should not be taken as the natural history of pyonephrosis; in the administrative cohorts described above, in-hospital mortality among decompressed patients with stone-associated sepsis ranged from under 2% to about 9%, depending on case definition and era [8,63]. Representative quantitative data underpinning these principles are summarized in Table 2.

Three sources of bias limit causal interpretation of these cohorts. The first is confounding by indication: clinicians decide whether, when, and how to drain according to illness severity, anatomy, and goals of care, so that patients who are never decompressed include both those too mildly ill to need drainage and those who were moribund or died before it could be arranged, and patients drained late differ systematically from those drained promptly—delay was more frequent after weekend admission and in non-white and lower-income patients [9]. The second is selection and immortal-time bias: patients must survive long enough to undergo a late procedure, deaths before any procedure are counted in the non-decompressed group, and only the most recent analysis addressed this with landmark and time-varying methods [63]; in addition, cohort entry depends on diagnosis and procedure codes that may misclassify both infection and drainage, and the timing is recorded only by calendar day. The third is residual confounding: administrative datasets contain no hemodynamic variables, lactate, organ-failure scores, microbiology, or antimicrobial timing, so that adjustment for demographics and comorbidity cannot fully equalize prognosis. Because a randomized comparison of drainage with no drainage would be unethical, the strong guideline recommendation for urgent decompression rests on physiological rationale, on the consistency and magnitude of these associations, and on clinical experience rather than on experimental proof, and the time threshold beyond which delay becomes harmful remains undefined.

Prognostic factors beyond timeliness of drainage include the severity of hydronephrosis at presentation, baseline renal function, the presence and severity of diabetes mellitus or other immunosuppression, overall sepsis severity, and the underlying cause, with malignant obstruction generally carrying a worse prognosis than calculous disease. Renal function not infrequently improves after decompression, attributed to restored perfusion, resolution of pressure-related tubular dysfunction, and improved antimicrobial penetration into the affected kidney—an instructive illustration, for the wider kidney disease community, of just how much apparently fixed renal impairment can in fact be reversible once its obstructive and infective drivers are removed, and of why the salvage-versus-nephrectomy decision (see Section 10.6) should be deferred until this recovery has had time to manifest. Conversely, each episode of pyonephrosis that goes unrecognized, is inadequately drained, or recurs may plausibly represent a discrete insult to nephron mass. Although no pyonephrosis-specific longitudinal data exist, it seems prudent—by analogy with other causes of AKI [32,33]—to offer patients with a history of pyonephrosis, particularly bilateral, recurrent, or xanthogranulomatous disease, longer-term follow-up of renal function.

## 12. Recent Advances and Future Directions

Imaging research in pyonephrosis has moved in recent years from qualitative pattern recognition toward quantitative and computational approaches. Beyond the Hounsfield-unit thresholds discussed in Section 9.2, a clinical–radiomic nomogram has been developed that combines a radiomic signature of 14 CT texture features extracted from the region of pelvicalyceal dilatation with independent clinical predictors (serum cystatin C, collecting-system Hounsfield unit, history of ipsilateral urological surgery, and urine culture) to predict the presence of pyonephrosis before surgery, achieving an area under the curve of 0.96 in a validation set and outperforming both the clinical and the radiomic model alone [68]. Machine-learning decision-support models built on routine clinical and laboratory inputs (for example, leukocyte and neutrophil counts, urine leukocytes, urine culture, and stone characteristics) have likewise been proposed to flag patients with calculous obstructive pyonephrosis at the point of presentation, potentially allowing earlier triage to urgent decompression before overt sepsis develops [69,70]. These models remain, at present, single-center and retrospective, and require external, prospective validation before they can be recommended for routine clinical use, but they point toward a future in which the morphological diagnosis of pyonephrosis is supported by automated, quantitative image analysis rather than by visual impression and a single manually placed region of interest alone—and, by extension, toward earlier identification of a potentially reversible cause of AKI before irreversible parenchymal loss has occurred.

In parallel, minimally invasive and robotic-assisted nephrectomy techniques continue to be refined for the inflammatory, densely adherent kidney characteristic of chronic pyonephrosis and xanthogranulomatous pyelonephritis, extending the benefits of a minimally invasive approach to a population historically thought to require open surgery, while antimicrobial stewardship principles—culture-directed, collecting-system-based sampling and prompt de-escalation—have become correspondingly more important as resistance among uropathogens continues to rise. Biomarker- and time-to-decompression-based triage, of the kind proposed using procalcitonin and related early warning parameters [41], represents a further active area of investigation aimed at more precisely identifying, among the broad population of patients with obstructive uropathy, the subset who require truly emergent rather than merely urgent decompression.

## 13. Synthesis and Limitations of the Evidence

Much of the literature specific to pyonephrosis, as distinct from the broader category of obstructive, infected urolithiasis, consists of retrospective cohorts and case series rather than randomized trials; the randomized comparisons of drainage modality reviewed in Section 10.2 enrolled patients with obstructing calculi and clinical signs of infection, urosepsis, or SIRS [24,59,60], stone-induced hydronephrosis irrespective of infection [25], or obstructing urolithiasis with any indication for drainage [61], rather than a pyonephrosis-specific cohort defined by aspirated pus, and their findings should be extrapolated to pyonephrosis proper with this distinction in mind. The debate over percutaneous nephrostomy versus retrograde ureteral stenting remains genuinely unresolved at the level of high-quality evidence: most randomized trials and the pooled analyses have detected no significant differences on clinical recovery endpoints, and one trial found faster defervescence after percutaneous nephrostomy; confidence intervals are wide, heterogeneity is substantial, and—apart from one non-inferiority trial in a population of which only about one-third had infection—no design was capable of demonstrating equivalence, while large observational, non-randomized cohorts report worse outcomes with percutaneous nephrostomy, a discrepancy most plausibly explained by confounding by indication rather than a true difference in efficacy, but not yet resolved by an adequately powered trial in infected, obstructed systems. Diagnostic-accuracy estimates for both ultrasonography and Hounsfield-unit CT quantification are similarly heterogeneous across studies, reflecting differences in patient selection, the definition of pyonephrosis used as the reference standard (radiological impression versus confirmed pus at aspiration), region-of-interest placement technique, and scanner protocol; readers should therefore treat any single sensitivity, specificity, or threshold value cited in this review as representative of the study from which it derives rather than as a universally applicable diagnostic cut-off. Mortality figures likewise vary substantially according to the era, healthcare setting, and severity of the underlying cohort studied, with historical and resource-limited series reporting considerably higher mortality than contemporary, promptly drained cohorts in well-resourced settings. Finally, terminological inconsistency between “pyonephrosis” and “infected hydronephrosis” across the literature complicates direct comparison between studies and should be considered when interpreting the pooled figures presented throughout this review; the long-term renal-functional and CKD-related consequences of pyonephrosis specifically have not, to our knowledge, been studied in dedicated longitudinal cohorts—the mechanistic argument advanced in Section 6.1 is therefore extrapolated from experimental models of urinary tract obstruction rather than from pyonephrosis-specific outcome data—and this represents a clear gap for future research relevant to kidney disease outcomes. The associations between decompression, its timing, and mortality are subject to confounding by indication, selection and immortal-time bias, and residual confounding analyzed in Section 11. In addition, because this is a narrative review, study selection was not exhaustive, no formal study-level risk-of-bias assessment or quantitative synthesis was performed, and the certainty ratings in Table 3 are the authors’ consensus judgments (Section 2.5).

Table 3 summarizes the principal conclusions of this review, the populations in which the supporting evidence was generated, and our rating of the certainty of each conclusion (Section 2.5). No conclusion reaches high or moderate certainty: most rest on small single-center diagnostic series, on randomized trials conducted in adjacent populations, or on observational associations in administrative data, and the guideline recommendations that underpin current management are accordingly based more on physiological rationale and clinical consensus than on direct experimental evidence in confirmed pyonephrosis.

## 14. Conclusions

Pyonephrosis exemplifies, perhaps more clearly than any other urological emergency, the transformation of a millennia-old, frequently fatal disease into one that is now, in most instances, promptly recognized and safely resolved—and, with that transformation, the conversion of what was once an almost inevitable route to renal destruction into an often reversible cause of AKI. Its diagnosis has become a substantially morphological one, resting on the sonographic identification of echogenic, dependent debris within a dilated collecting system, supported, where available, by higher attenuation of that fluid at CT and by restricted diffusion at MRI—findings that complement but do not replace clinical assessment, and whose reported thresholds are protocol- and population-dependent rather than validated cut-offs; the schematics and the case from our own practice presented in this review show how heterogeneous and how advanced that morphology can become. Its management rests on a small number of widely accepted principles—recognition of urosepsis, urgent decompression by percutaneous nephrostomy or retrograde ureteral stenting, culture of collecting-system urine, deliberately delayed definitive treatment of the underlying cause, and nephrectomy reserved for the truly destroyed kidney—that are strongly endorsed by guidelines but supported mainly by observational associations and low-certainty evidence, much of it generated in obstructive pyelonephritis or stone-associated sepsis rather than in confirmed pyonephrosis (Table 3). The choice between the two decompression techniques remains unresolved: the few small trials have given inconsistent results, and neither superiority nor equivalence has been established in infected systems. What has changed most decisively since Thomas Hillier’s improvised puncture of a hydronephrotic kidney in 1865 is not the biology of the disease itself but the speed, precision, and safety with which it can now be identified and drained—the achievement that appears to have converted pyonephrosis from an immemorial cause of death and of renal destruction into a contemporary, generally survivable emergency. Whether, and how often, timely drainage also protects long-term renal function is a question that dedicated longitudinal studies have yet to answer.

## Figures and Tables

**Table 1:** Randomized trials, meta-analyses, and observational cohorts comparing percutaneous nephrostomy (PCN) and retrograde ureteral stenting for urgent decompression of the obstructed upper urinary tract, with the population enrolled in each study; none was restricted to confirmed pyonephrosis.

Study	Design and Population	n	Principal Finding(s)	Ref.
Pearle et al. (1998)	RCT; obstructing ureteral calculi with temperature ≥ 38 °C and/or WBC ≥ 17,000/mm^3^; grossly purulent urine at drainage in 6/42 (category C)	42	No significant difference between PCN and retrograde catheterization in time to normal temperature (2.3 vs. 2.6 d) or WBC (2.0 vs. 1.7 d) or in length of stay (4.5 vs. 3.2 d); sized to detect a 1 d difference (17 per group), power < 80% by the authors’ account, no CIs reported, no equivalence or non-inferiority design; one technical failure (PCN arm), rescued by retrograde catheterization; PCN less costly, retrograde catheterization shorter procedure/fluoroscopy time (32.7 vs. 49.2 min; 5.1 vs. 7.7 min); collecting-system cultures positive more often after PCN (61.9% vs. 19.1%); back discomfort and parenteral narcotic use greater with PCN; neither modality superior for clinical recovery.	[24]
Mokhmalji et al. (2001)	RCT; stone-induced hydronephrosis requiring diversion, not restricted to infection (category E)	40	Retrograde stent placement failed in ~20% (more often with proximal stones and age >60 y), with rescue by PCN; PCN succeeded in 100%; IV antibiotics needed >5 d in 0% (PCN) vs. 64% (stent; not significant); quality of life improved with PCN, deteriorated with stenting; PCN favored overall by the authors, especially with fever, in males, and in younger patients; not designed to test equivalence.	[25]
Xu et al. (2021)	RCT (single center); upper urinary tract stones with urosepsis (category C)	65	Time to normal temperature shorter after PCN (3.0 vs. 5.0 d, as extracted in [58]) and postoperative C-reactive protein lower (*p* < 0.05); 7 patients with failed retrograde stenting excluded from the analysis; PCN favored by the authors, especially with severe inflammation and fever.	[59]
Lu et al. (2023)	RCT (single center); ureteral stones with SIRS (category C)	150	No significant difference between PCN (n = 78) and stent (n = 72) in post-decompression urosepsis (15.4% vs. 22.2%; *p* = 0.283), time to normalization of vital signs (2.35 vs. 2.51 d; *p* = 0.072), or length of stay (7.92 vs. 8.37 d; *p* = 0.215); purulent fluid at drainage and higher procalcitonin associated with progression to urosepsis.	[60]
Hendriks et al. (2025)	Non-inferiority RCT (11 hospitals); obstructing urolithiasis with an indication for drainage—pain 73%, renal impairment 62%, infection 35% (category E)	204	PCN non-inferior to stent for time to clinical recovery (1.7 vs. 1.5 d; MD 0.3 d, 95% CI −0.2 to 0.7; margin 1 d); crossover for technical failure in 11/101 (PCN) vs. 2/103 (stent); reintervention within 3 months in 17% vs. 6.4% (per protocol); adverse events similar.	[61]
Moon et al. (2024)	Meta-analysis (8 studies: 3 RCTs and 5 retrospective cohorts); severe urinary tract infection with obstructive urolithiasis (categories C–D)	—	No significant difference in procedure success rate (OR 0.86, 95% CI 0.34–2.20), time to normalization of temperature (MD −0.74 d, 95% CI −1.69 to 0.21; I^2^ = 96%) or WBC (MD 0.46 d, 95% CI −0.30 to 1.21; I^2^ = 85%), or length of stay (MD 0.45 d, 95% CI −2.78 to 3.68; I^2^ = 96%); wide intervals do not establish equivalence; choice between PCN and stenting depends mainly on urologist/patient preference.	[58]
Zul Khairul Azwadi et al. (2021)	Systematic review/meta-analysis (2 RCTs + 5 CCTs, 667 patients); acute upper obstructive uropathy, not restricted to infection (category E)	—	No significant difference in improvement of septic parameters, failure rates, or EQ-5D quality of life; PCN associated with less post-procedural hematuria/dysuria (favored by the authors on this basis), at the cost of longer hospitalization (MD 1.82 d, 95% CI 0.79–2.85).	[62]
Sammon et al. (2013)	Retrospective cohort (USA Nationwide Inpatient Sample, 1999–2009); infected urolithiasis identified from diagnosis codes (category D)	396,385 (weighted)	Incidence of hospitalization with infected urolithiasis about twice as high in women as in men; sepsis/severe sepsis increased over time; PCN use declined and, in propensity-matched analysis, was associated with higher odds of sepsis (OR 1.63, 95% CI 1.52–1.74), severe sepsis (OR 2.28, 95% CI 2.06–2.52), prolonged stay (OR 3.18, 95% CI 3.01–3.34), and death (OR 3.14, 95% CI 2.13–4.63) in this non-randomized dataset (propensity score without any measure of severity), most plausibly reflecting confounding by indication.	[28]
Dogha et al. (2026)	Retrospective cohort (USA National Inpatient Sample, 2016–2022); sepsis or septic shock with obstructing ureteral stones (category D)	9172	Overlap-weighted mortality 5.5% (PCN) vs. 2.8% (stent), OR 2.05 (95% CI 1.31–3.09); PCN patients more often transferred, comorbid, and on vasopressors or ventilation; difference regarded by the authors as hypothesis-generating (residual confounding by indication and feasibility).	[63]
Goldsmith et al. (2013)	Retrospective single-health-system cohort; obstructive urolithiasis with SIRS (category C)	130	Failed procedures 2.3% overall; PCN patients had larger stones (10 vs. 7 mm) and higher APACHE II scores (15 vs. 11); PCN associated with ICU admission (OR 3.23, 95% CI 1.24–8.41) and longer stay after adjustment for age, APACHE II score, and comorbidity.	[64]

PCN = percutaneous nephrostomy; RCT = randomized controlled trial; CCT = controlled clinical trial; WBC = white blood cell count; IV = intravenous; USA = United States of America; CI = confidence interval; MD = mean difference (PCN minus stent); OR = odds ratio; SIRS = systemic inflammatory response syndrome; ICU = intensive care unit; APACHE II = Acute Physiology and Chronic Health Evaluation II. Population categories (A–E) are defined in Section 4.

**Table 2:** Representative morphological and outcome data underpinning the imaging and management principles discussed in this review, with the population and reference standard in which each estimate was generated. Diagnostic thresholds are study-specific and have not been externally validated.

Parameter	Reported Value	Cohort, Population, and Reference Standard	Ref.
Sonographic sensitivity/specificity for pyonephrosis (internal echoes)	90%/97% (original series); 62% sensitivity on re-evaluation (specificity maintained at 100%); confidence intervals not reported	n = 73 cases, 92 kidneys (34 with clinically suspected infection); re-evaluation cohort; small single-center series	[43,44]
Study-specific CT attenuation thresholds for pyonephrosis (each derived and tested in the same cohort)	≥6.3 HU (sens. 71.6%/spec. 71.5%; AUC 0.76, 95% CI 0.66–0.85); ≥8.46 HU (sens. 68.4%/spec. 92.6%; CI not reported); ≥9.5 HU (sens. 71.4%/spec. 70.2%; AUC 0.613)	n = 122, mixed obstructive uropathy; reference standard: visual purulence at drainage; n = 159 (214 renal units); reference standard: needle aspiration and culture; n = 240, upper urinary tract stones	[6,10,48]
DWI-MRI apparent diffusion coefficient (×10^−3^ mm^2^/s); protocol-dependent, no threshold derived	Pyonephrosis 0.64 ± 0.35; hydronephrosis 2.98 ± 0.65	n = 12 (4 pyonephrosis, 8 hydronephrosis); microbiological confirmation	[52]
Retrograde stent placement failure rate	~20%, with rescue by PCN (more often with proximal stones and age >60); PCN success 100%	n = 40, RCT; stone-induced hydronephrosis, not restricted to infection (category E)	[25]
In-hospital mortality, decompressed vs. not decompressed (observational association)	8.82% vs. 19.2% (OR 2.6, 95% CI 1.9–3.7 for death without decompression, adjusted)	n = 1712; sepsis with ureteral calculi in administrative data (category D)	[8]
SIRS/sepsis after drainage, pyonephrosis vs. hydronephrosis	78.3% vs. 31.6%/52.2% vs. 13.1%	n = 122; pyonephrosis defined by visual purulence at drainage (category A)	[6]
Positive collecting-system urine culture, pyonephrosis vs. hydronephrosis	86.9% vs. 14.4%	n = 122; pyonephrosis defined by visual purulence at drainage (category A)	[6]
Predictors of pyonephrosis in renal calculus disease	Staghorn calculi, longer symptom duration, moderate-to-severe hydronephrosis, associated ipsilateral UPJ obstruction or ureteric calculus, multiple stones, non-functioning kidney; prior urological surgery (multivariate analysis)	n = 501 (91 pyonephrosis, 410 without); renal calculus disease	[27]

HU = Hounsfield unit; DWI-MRI = diffusion-weighted magnetic resonance imaging; SIRS = systemic inflammatory response syndrome; OR = odds ratio; CI = confidence interval; UPJ = ureteropelvic junction; AUC = area under the receiver operating characteristic curve; PCN = percutaneous nephrostomy; RCT = randomized controlled trial. Population categories (A–E) are defined in Section 4.

**Table 3:** Principal conclusions of this review, the populations in which the supporting evidence was generated, and the authors’ rating of the certainty of each conclusion.

Conclusion	Supporting Evidence (Design; Population)	Certainty	Main Reasons for Rating
Echogenic debris or internal echoes in a dilated collecting system support the diagnosis of pyonephrosis; their absence does not exclude it.	Two small single-center sonographic series in suspected or proven pyonephrosis [43,44].	Low	Small samples; inconsistent sensitivity (62–90%); confidence intervals not reported; dated technology.
Collecting-system fluid attenuation at non-contrast CT is, on average, higher in pyonephrosis than in uninfected hydronephrosis.	Single-center retrospective cohorts with reference standards ranging from visual purulence to aspirate or pelvic-urine culture (mainly categories A–B) [6,10,48,49,50,51].	Low	Direction consistent in most but not all studies; heterogeneous reference standards and measurement protocols; no external validation.
A specific HU threshold can be used as a diagnostic cut-off.	The same studies; proposed thresholds range from 0 to 9.5 HU.	Very low	Thresholds derived and tested in the same samples; AUC 0.61–0.79; protocol- and population-dependent.
Markedly restricted diffusion (low ADC) distinguishes pyonephrosis from hydronephrosis.	One series of 12 patients with microbiological confirmation [52].	Very low	Very small sample; no accuracy estimates or confidence intervals; ADC values are protocol-dependent.
Bladder or midstream urine culture frequently fails to identify the pathogen present in the obstructed collecting system, from which urine should therefore be cultured.	Small observational series in categories A–C and in obstructing ureteral stones [11,12,13,29]; guideline recommendations [38,39,40].	Low	Consistent direction across settings, but small, heterogeneous series; magnitude imprecise (about one-third to two-thirds).
Urgent decompression of the obstructed, infected system is associated with lower mortality.	Administrative cohorts of stone-associated sepsis (category D) [8,63]; strong guideline recommendations, graded as resting on Grade C evidence in the American guideline [38,39].	Low (for a causal effect)	Large and consistent associations, but confounding by indication, immortal-time bias, and residual confounding; indirect for confirmed pyonephrosis; randomized comparison not feasible.
Delayed decompression is associated with worse outcomes; the critical time threshold is undefined.	Administrative cohorts and one systematic review (categories C–D) [9,41,63].	Low	Observational; timing recorded by calendar day; thresholds inconsistent between studies.
PCN and retrograde stenting lead to similar clinical recovery; neither is proven superior or equivalent in infected systems.	Three RCTs in infected, obstructed systems (category C), two without significant differences and one favoring PCN for defervescence [24,59,60]; one RCT in stone-induced hydronephrosis (category E) [25]; one non-inferiority RCT in obstructing urolithiasis, 35% infected [61]; meta-analyses [58,62]; confounded observational cohorts [28,63,64].	Low	Inconsistency between trials; imprecision; heterogeneity; indirectness (no trial in confirmed pyonephrosis). The American guideline grades the evidence that either modality may be used as Grade A; the lower rating here refers to pyonephrosis specifically.
Technical failure and reintervention rates differ between the two techniques and depend on the setting.	Four RCTs [24,25,59,61].	Low	Direction inconsistent between trials (stent failure 0–20%; PCN failure 0–12%); small numbers of events.
Definitive treatment of the obstructing cause should be deferred until infection is controlled.	Guideline recommendations [38,39]; one RCT of emergent ureteroscopy in stable septic patients [65].	Low	Largely consensus-based; single trial in a selected population.
Pyonephrosis contributes to long-term CKD.	Experimental models of obstruction and the general AKI-to-CKD literature [30,31,32,33]; cross-sectional and short-term clinical data [27,47].	Very low	No pyonephrosis-specific longitudinal data; indirect evidence only.
Radiomic and machine-learning models improve the preoperative identification of pyonephrosis.	Single-center retrospective model-development studies [68,69,70].	Very low	No external or prospective validation.

Certainty ratings are the authors’ consensus judgments, informed by the GRADE domains (Section 2.5); they do not derive from a formal GRADE assessment. Population categories: A = confirmed pyonephrosis; B = infected hydronephrosis; C = obstructive pyelonephritis; D = stone-associated sepsis or infected urolithiasis identified from administrative codes; E = obstruction irrespective of infection (Section 4). ADC = apparent diffusion coefficient; AKI = acute kidney injury; AUC = area under the receiver operating characteristic curve; CKD = chronic kidney disease; CT = computed tomography; HU = Hounsfield unit; PCN = percutaneous nephrostomy; RCT = randomized controlled trial.

## Data Availability

No new data were created or analyzed in this study. Data sharing is not applicable to this article.

## Abbreviations

The following abbreviations are used in this manuscript:

ADC	Apparent diffusion coefficient
AKI	Acute kidney injury
AUA	American Urological Association
AUC	Area under the receiver operating characteristic curve
CI	Confidence interval
CKD	Chronic kidney disease
CT	Computed tomography
DMSA	Dimercaptosuccinic acid
DWI	Diffusion-weighted imaging
EAU	European Association of Urology
EPN	Emphysematous pyelonephritis
ESBL	Extended-spectrum beta-lactamase
GRADE	Grading of Recommendations Assessment, Development and Evaluation
HU	Hounsfield unit
IVU	Intravenous urography
KIM-1	Kidney injury molecule-1
MAG3	Mercaptoacetyltriglycine
MRI	Magnetic resonance imaging
NGAL	Neutrophil gelatinase-associated lipocalin
OR	Odds ratio
PCN	Percutaneous nephrostomy
QUADAS-2	Quality Assessment of Diagnostic Accuracy Studies-version 2
qSOFA	Quick Sequential Organ Failure Assessment
RCT	Randomized controlled trial
RoB 2	Revised Cochrane risk-of-bias tool for randomized trials
ROBINS-I	Risk Of Bias in Non-randomized Studies of Interventions
SANRA	Scale for the Assessment of Narrative Review Articles
SIRS	Systemic inflammatory response syndrome
XGP	Xanthogranulomatous pyelonephritis
